# Homocysteine-targeting compounds as a new treatment strategy for diabetic wounds via inhibition of the histone methyltransferase SET7/9

**DOI:** 10.1038/s12276-022-00804-1

**Published:** 2022-07-20

**Authors:** Guodong Li, Dan Li, Chun Wu, Shengnan Li, Feng Chen, Peng Li, Chung-Nga Ko, Wanhe Wang, Simon Ming-Yuen Lee, Ligen Lin, Dik-Lung Ma, Chung-Hang Leung

**Affiliations:** 1 State Key Laboratory of Quality Research in Chinese Medicine, Institute of Chinese Medical Sciences, University of Macau, Macao, China; 2 Zhuhai UM Science and Technology Research Institute, Zhuhai, 519031 China; 3 Department of Chemistry, Hong Kong Baptist University, Kowloon Tong, Hong Kong, China; 4 Institute of Medical Research, Northwestern Polytechnical University, Xi’an, Shaanxi China; 5 Department of Biomedical Sciences, Faculty of Health Sciences, University of Macau, Macao, China

**Keywords:** Drug discovery, Diabetes complications

## Abstract

In hypoxia and hyperglycemia, SET7/9 plays an important role in controlling HIF-1α methylation and regulating the transcription of HIF-1α target genes, which are responsible for angiogenesis and wound healing. Here, we report the Ir(III) complex **Set7_1a** bearing acetonitrile (ACN) ligands as a SET7/9 methyltransferase inhibitor and HIF-1α stabilizer. Interestingly, **Set7_1a** could engage SET7/9 and strongly inhibit SET7/9 activity, especially after preincubation with homocysteine (Hcy), which is elevated in diabetes. We hypothesize that **Set7_1a** exchanges ACN subunits for Hcy to disrupt the interaction between SET7/9 and SAM/SAH, which are structurally related to Hcy. Inhibition of SET7/9 methyltransferase activity by **Set7_1a** led to reduced HIF-1α methylation at the lysine 32 residue, causing increased HIF-1α level and recruitment of HIF-1α target genes that promote angiogenesis, such as VEGF, GLUT1, and EPO, in hypoxia and hyperglycemia. Significantly, **Set7_1a** improved wound healing in a type 2 diabetic mouse model by activating HIF-1α signaling and downstream proangiogenic factors. To our knowledge, this is the first Hcy-targeting iridium compound shown to be a SET7/9 antagonist that can accelerate diabetic wound healing. More importantly, this study opens a therapeutic avenue for the treatment of diabetic wounds by the inhibition of SET7/9 lysine methyltransferase activity.

## Diabetes: A new way to heal wounds

Animal trials have demonstrated the potential of a new drug strategy to heal the wounds associated with diabetes, especially in the feet,which often lead to chronic damage, sometimes treatable only by amputation. Leung CH and Lin L at the University of Macau, China, and Ma DL at the Hong Kong Baptist University tested the new therapy on a mouse model of type 2 diabetes. The treatment uses a homocysteine-targeting metal complex that inhibits a key enzyme SET7/9 involved in the processes that cause diabetic wounds. The treatment activated a molecular signalling cascade involved in generating the new blood vessels needed for wounds to heal. It could help address the urgent need for better treatments for this serious problem.

## Introduction

Patients with diabetes have high amputation rates due to chronic and nonhealing wounds and the lack of available medical tools^1^. Becaplermin, which is recombinant platelet-derived growth factor-BB (rhPDGF-BB), is the first FDA-approved product for wound healing. However, the use of becaplermin has been linked with an enhanced risk of cancer mortality^2^. Bioengineered skin-care products, such as *Apligraf* and *Dermagraft*, have been approved by the FDA for diabetic foot ulcer (DFU) treatment^3^. However, compared with the standard of care in terms of complete wound closure, these products show limited superiority^3^. Thus, effectively treating diabetic wounds is still a major challenge in wound care^4^.

Hyperglycemia and hypoxia play key roles in the pathophysiological outcomes of diabetes due to the dysfunctional response of tissues to insufficient oxygen concentrations^5^. The transcription factor hypoxia-inducible factor-1 alpha (HIF-1α) is considered a key mediator of wound healing. HIF-1α stimulates angiogenesis by binding to the hypoxia response element (HRE) of target genes, including stromal cell-derived factor 1 (SDF-1), vascular endothelial growth factor (VEGF), and angiopoietin 2. This molecule also transcriptionally upregulates the expression of other key players in the wound healing process^6^. In external wounds, HIF-1α can also mediate the inflammatory response through its regulation of the glycolytic metabolic switch. The enhancement of angiogenesis by increased HIF-1α is critical for wound healing, as it contributes to the delivery of both oxygen and nutrients to the wound site. However, hyperglycemia suppresses HIF-1α levels, and only very low levels of HIF-1α protein are detected in diabetic wounds^7^. Hence, hyperglycemia-dependent destabilization and suppression of HIF-1α is a central pathogenic mechanism in diabetic wounds^8^. This finding suggests that stabilization of HIF-1α in hyperglycemia could be a potential strategy for improving defective wound healing in diabetic mellitus.

In normoxia, the hydroxylation of HIF-1α by prolyl hydroxylase domain proteins (PHDs) in the cytoplasm promotes HIF-1α degradation via the Cullin 2 (CUL2)-E3 ubiquitin ligase complex^9^. PHD inhibitors, including FG-2216, BAY-853934, FG-4592, ethyl 3,4-dihydroxybenzoate (EDHB), and GSK1278863, have hence been studied as stabilizers of HIF-1α^10^. Although PHD inhibitors have entered clinical trials for angiogenesis-related diseases, they are limited by low target selectivity and negative side effects^11^. For instance, FG-2216 was linked to liver abnormalities in a phase 2 clinical trial, and one patient developed fatal hepatic necrosis^12^. Alternatively, blocking the downstream interaction of HIF-1α and Von Hippel-Lindau tumor suppressor (VHL) protein, which is a negative regulator of HIF-1α, is another potential strategy to inhibit HIF-1α degradation. For example, VH298, the most effective in vitro VHL-HIF-1α protein-protein interaction (PPI) inhibitor described to date, has been reported to selectively stabilize hydroxylated HIF-α and promote enthesis healing and wound healing in vivo^13–15^. In addition to VH298, our group recently reported a small molecule VHL-HIF-1α PPI inhibitor as a promising agent for wound healing in vivo^16^. Although several small-molecule HIF-1α stabilizers for treating wound healing have been reported in the literatures, none have yet entered clinical trials for treating diabetic wounds. Notably, hyperglycemia was able to decrease HIF-1α stabilization even in the presence of the PHD inhibitor EDHB in both hypoxia and normoxia, suggesting that mechanisms other than proline hydroxylation may be involved in the regulation of HIF-1α protein turnover in diabetes^17,18^.

Histone methyltransferase (HMT) SET domain containing lysine methyltransferase 7 (SET7/9), also known as KMT7, SET7, SETD7, or SET9, was one of the first protein lysine methyltransferases to be discovered. In diabetes, enhanced activity of SET7/9 in peripheral blood mononuclear cells has been detected and was correlated with epigenetic modifications. Dysfunction of SET7/9 has therefore been implicated in defective wound healing, ischemia, chronic anemia, and cardiovascular disease. SET7/9 contributes to hyperglycemia-induced inflammation in cell culture and animal models^19^. Macrophages of diabetic mice displayed increased expression of inflammatory genes as well as enhanced SET7/9 recruitment^20,21^. Additionally, SET7/9 mediates glucose stimulation of vascular endothelial cells via both histone and nonhistone mechanisms^22^. More importantly, studies have shown that sustained SET7/9 vascular gene expression was enhanced in diabetes as a response to hyperglycemia in vascular endothelial cells^23,24^.

The histone methyltransferase SET7/9-dependent methylation and histone demethylase LSD1-dependent demethylation cycle regulates HIF-1α stability in both VHL-independent and proline hydroxylation manners, indicating that SET7/9 plays an important function in controlling the level of HIF-1α and regulating HIF-α transcriptional activity^25,26^. The methylation of HIF-1α by SET7/9 leads to protein degradation of HIF-1α, thus diminishing the transcription of HIF-1α target genes, including those responsible for angiogenesis and wound healing^12^. Therefore, the stabilization of HIF-1α by inhibiting SET7/9 function and antagonizing HIF-1α methylation is a possible strategy to enhance diabetic wound healing. To date, several attempts have been made to identify SET7/9 inhibitors, including S-adenosyl-L-methionine (SAM, also known as AdoMet) competitive inhibitors (e.g., AAM-1, DAAM-3, sinefungin)^27,28^, histone lysine competitive inhibitors (e.g., (R)-PFI-2)^29^, and other inhibitors (e.g., GSK343, GSK926, SETin-1, DC-S238, DC-S239)^30–32^. However, many of the reported SET7/9 inhibitors are multitarget inhibitors with low potencies^31^.

Metal-based compounds with high structural variety and easily adjustable electronic and steric characteristics possess several promising advantages as protein or enzyme inhibitors in comparison to organic compounds^33–39^. Metal complexes can participate in ligand exchange reactions with biomolecules to exert their biological effects^40,41^. Herein, we report the application of the reported Ir(III) complex **Set7_1a** as a potent inhibitor of SET7/9. **Set7_1a** induces the accumulation of HIF-1α, especially after preincubation with homocysteine (Hcy), an elevated factor in diabetes, to upregulate the expression of HIF-1α target proteins *in cellulo* and in vivo. Mechanistic studies suggested that **Set7_1a** bearing acetonitrile (ACN) ligands could undergo ligand exchange reactions with Hcy to disrupt the interaction between SET7/9 and SAM, which are structurally related to Hcy. This study not only provides critical mechanistic insight into the potential protective effect of **Set7_1a** against HIF-1α degradation by inhibiting SET7/9 function and antagonizing HIF-1α methylation in hypoxia and hyperglycemia but also validates the feasibility of targeting SET7/9 methyltransferase activity for treating diabetic wounds. We also anticipate that Hcy-targeting **Set7_1a** could be employed as a new scaffold for the future development of more selective and potent antagonists of SET7/9 as clinical therapeutics for diabetic wound healing.

## Materials and methods

### Materials, antibodies, and chemicals

SET7/9 polyclonal rabbit antibody (NBP2-99744), HIF-1α antibody (NB100-479) and VEGF antibody (NB100-664) were purchased from Novus Biologicals (Littleton, CO, USA). VHL antibody (GTX101087) was purchased from GeneTex (Alton Pkwy Irvine, CA, USA). PHD2 antibody (A300-322A) was purchased from Bethyl Laboratories, Inc. (Montgomery, Texas, USA). A VEGF ELISA kit (EHC108) was purchased from NeoBioscience (Shenzhen, Guangdong, China). The sequences of RNA interference oligonucleotides are as follows^42^:

SET7/9 siRNA: sense, 5’- GGGCACCUGGACGAUGACGGA-3’, antisense, 5’- UCCGUCAUCGUCCAGGUGCCC-3’.

Ctr siRNA: sense, 5’- UUCUCCGAACGUGUCACGU-3’, antisense, 5’- ACGUGACACGUUCGGAGAA-3’.

### Fluorescence polarization

The fluorescence polarization assay was performed based on the instructions of the SET7/9 SAM-Screener^TM^ Assay Kit. Briefly, 10 μL of SET7/9 (human recombinant) assay enzyme was incubated with 5 μL of positive control or negative control or complexes for 15 minutes at room temperature in a 384-well solid plate (low volume; black; 400093; Cayman Chemicals, Ann Arbor, MI, USA). Then, 5 μL of the reconstituted SAM-binding site probe was added to each well for 30 minutes at room temperature. The signals were recorded using a SpectraMax M5 microplate reader (Ex = 575 nm, Em = 620 nm). A two-sided t test was used to calculate *P* values.

### Coimmunoprecipitation

Hyperglycemia-induced HUVECs (1 × 10^6^ cells/well) were treated with the most promising complex, **Set7_1a**, or DMSO for 24 h under hypoxia. One hundred micrograms of the collected cell lysate was incubated with 10 μg of SET7/9 antibody in 500 μL of cell lysis buffer at 4 °C for 12 h. The proteins were immunoprecipitated using magnetic beads. The levels of the proteins coprecipitated by magnetic beads were analyzed with anti-SET7/9, anti-methyl lysine, and anti-HIF-1α antibodies and then visualized using ECL Western Blotting Detection Reagent (GE Healthcare, Madison, WI, USA).

### In vitro scratch assay

Briefly, 5 × 10^5^ hyperglycemia-induced HUVECs were seeded in 6-well plates under hypoxic conditions. Then, scratches were generated using a 200 μL plastic pipette tip when cells were grown to a confluent monolayer, and the cells were then washed with a medium containing 0.5% FBS three times. After treatment with compounds in hypoxia, images of the wounded monolayer of HUVECs were taken at 0, 12, 24, and 36 h using a bright-field inverted microscope (Nikon, Japan).

### Animal experiments

Male C57BL/6 J mice were housed in the animal facility of the University of Macau and maintained at 23 ± 1 °C under a 12 h light/12 h dark cycle with 50% humidity and free access to water and food. Six- to eight-week-old mice were randomly divided into two groups. One group of mice (DM) was fed a high-fat diet (HFD, 60% calories from fat, Trophic Animal Feed High-Tech Co., Nantong, Jiangsu, China) for 8 weeks and then intraperitoneally injected with streptozotocin (STZ, 40 mg/kg body weight, 0.1 M citrate buffer, pH 4.5, Sigma-Aldrich, St. Louis, MO, USA) daily for 7 days. The other group of mice (NC) was fed a regular chow diet (Guangdong Medical Lab Animal Center, Guangzhou, Guangdong, China) for 8 weeks and then intraperitoneally injected with the same volume of citrate buffer. Three days after the last STZ injection, the mice in the DM group were fasted for 6 h, and then, the blood glucose levels were measured by a One-Touch Ultra glucometer (Lifescan, Milpitas, CA, USA). The mice with blood glucose between 15.0 and 28 mmol/L, accompanied by manifestations of polydipsia, polyuria and polyphagia, were considered to be mimic type 2 diabetic mice and used in the following experiments. The NC and DM mice were randomly allocated into two groups. The vehicle and **Set7_1a** groups were intraperitoneally injected with vehicle (PEG 400: distilled water = 6:4, v/v) and **Set7_1a** (50 mg/kg in PEG400 solution), respectively, every other day for 8 days.

### ICP-MS study

Fifty microliters of the skin or plasma samples harvested after 8 days from the control and treatment groups were completely digested in 5 mL of a 68% HNO_3_:H_2_O_2_ (v/v = 4:1) solution. The iridium content in skin samples was determined by ICP-MS with an iridium standard solution (Sigma-Aldrich, St Louis, MO, USA) and calculated as pg[iridium]/μg[protein], and data from plasma samples were calculated as pg[iridium]/μL[plasma].

### Assessment of skin perfusion

Regional skin perfusion at the wound area of mice was measured using a laser Doppler imager (PeriCam PSI System, Perimed, Sweden).

### Statistical analysis

The number of samples or animals is specified in the figure for each experiment. One-way analysis of variance (ANOVA) was performed to assess the significant differences between groups unless otherwise noted. Significant differences were claimed when *P* < 0.05.

Detailed information about other experiments is included in the Supporting Information file.

## Results

### Screening of small molecules as SET7/9 inhibitors

**Fig. 1:**
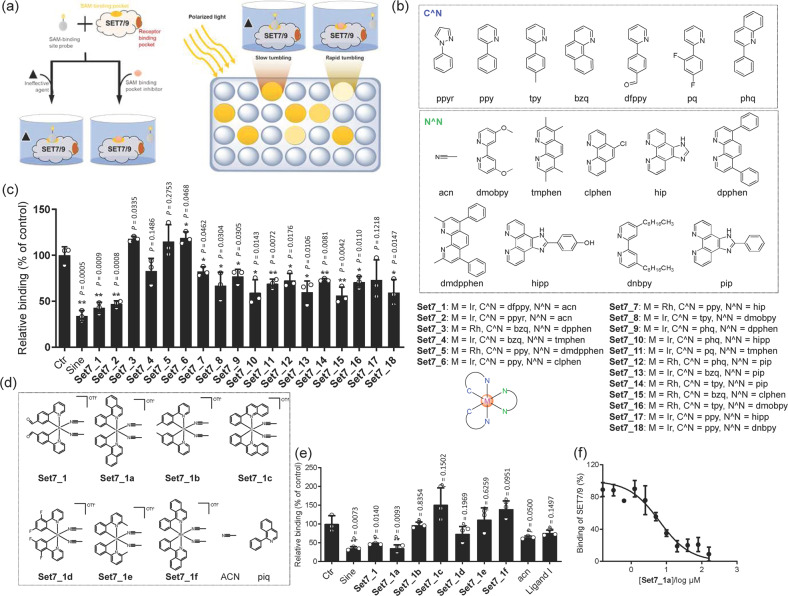
Screening of small molecules as SET7/9 inhibitors. **a** Schematic diagram of the FP assay to monitor the binding ability of the complexes to SET7/9. **b** Chemical structures of the in-house Ir(III) and Rh(III) complexes. Complexes **1**–**2** are OTf^–^ salts, and complexes **3**–**18** are PF_6_^–^ salts. **c** Displacement of a SAM binding site probe from SET7/9 by complexes **1**–**18** and sinefungin (Sine) as determined by an FP assay. **d** Chemical structures of the focused library of metal complexes **Set7_1a**–**Set7_1f** (racemates) and the ACN and piq ligands. **e** Engagement of the SAM binding site in SET7/9 by complexes **Set7_1a**–**Set7_1f** and isolated ligands as determined by an FP assay. **f** Dose-dependent effect of the complex **Set7_1a** in the FP assay. IC_50_ = 6.31 μM. ^*^P < 0.05, ^**^P < 0.01 *vs*. the control (Ctr) group.

As a protein lysine methyltransferase (PLMT/PKMT), SET7/9 catalyzes the methylation of the epsilon amino group in lysine residues using SAM as a cofactor, while SAM is converted into the Hcy derivative S-adenosyl-L-homocysteine (SAH). In this study, a fluorescence polarization (FP) assay was performed to investigate the binding of the transition metal complexes to SET7/9 in the presence of a small molecule fluorescent probe that selectively binds to the SAM-binding pocket in SET7/9 (Fig. 1a). An in-house library of cyclometalated Ir(III)/Rh(III) metal complexes (**Set7_1**–**Set7_18)** with varying ligands was initially utilized to explore their ability to inhibit SET7/9 (Fig. 1b). Among the 18 potential metal complexes, the Ir(III) complexes **Set7_1** and **Set7_2** showed significant displacement of the SAM binding site probe (Fig. 1c), with slightly lower potency than the positive control, sinefungin (5’-deoxy-5’-(1,4-diamino-4-carboxybutyl)adenosine) (Supplementary Fig. 1), a previously reported SET7/9 inhibitor^28^. The complexes **Set7_1** and **Set7_2** contain different C^N ligands, but both possess two ACN ligands, suggesting that the ACN group might play a role in inhibiting the interaction of SAM-SET7/9.

Inspired by the promising results from the complexes **Set7_1** and **Set7_2**, an ACN-containing focused library of 5 cyclometalated Ir(III) complexes (**Set7_1a**–**Set7_1e**) (Fig. 1d) was designed and synthesized. The Ir(III) complex **Set7_1a**, bearing two 1-phenylisoquinoline (piq) C^N ligands, showed the highest displacement of the SAM binding site probe from SET7/9 (Fig. 1e). This result suggested that the piq ligand is superior to other C^N ligands, such as the 2-(*p*-tolyl)pyridine (tpy) (**Set7_1b**), 2-phenylquinoline (pq) (**Set7_1c**), 2-(2,4-difluorophenyl)pyridine (dFppy) (**Set7_1d**) and 2-methyl-6-phenylpyridine (mppy) (**Set7_1e**) ligands (Fig. 1e). Significantly, the Ir(III) complex **Set7_1a** also showed higher potency than the Rh(III) congener **Set7_1** **f**, indicating that the metal ion is crucial for biological activity. Finally, the isolated N^N and C^N ligands of complex **Set7_1a**, piq and ACN, showed negligible engagement of the SAM binding site in SET7/9 in the FP assay. This finding indicates that the Ir(III) center plays a key role in organizing the ligands into a biologically active arrangement. To further investigate the potency of **Set7_1a** in modulating the SAM-SET7/9 interaction, we performed dose-response assays of **Set7_1a** (Fig. 1f and Supplementary Fig. 2). The results demonstrated that the complex **Set7_1a** exhibited dose-dependent displacement of the SAM binding site probe from SET7/9 with an IC_50_ value of approximately 6.31 μM, which was comparable to sinefungin (6.17 μM).

### Set7_1a disrupts the SAM-SET7/9 interaction by engaging SET7/9

**Fig. 2:**
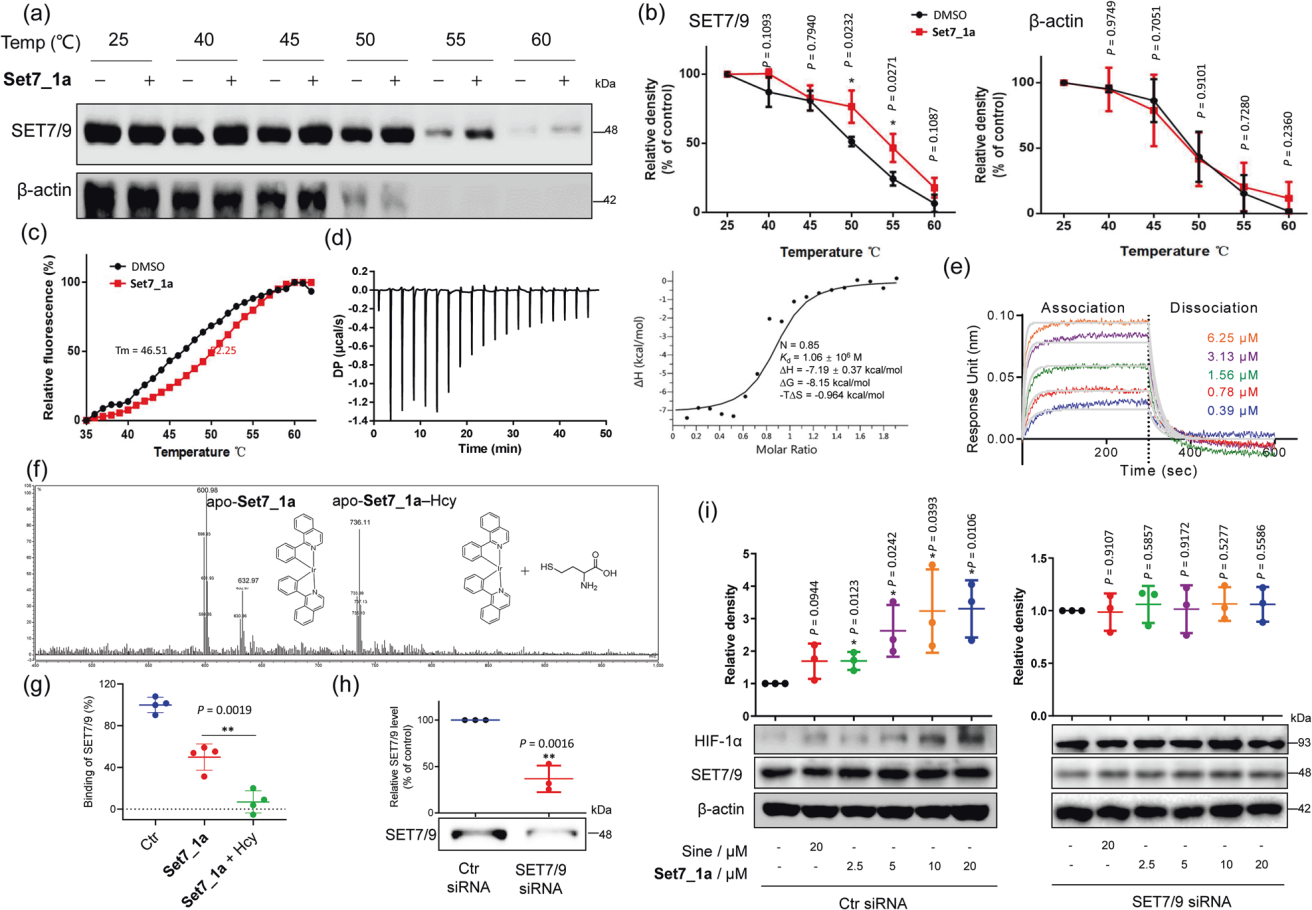
The Set7_1a complex disrupts the SAM–SET7/9 interaction by engaging SET7/9. **a**, **b** Western blot analysis to evaluate the effect of the **Set7_1a complex** (10 μM) on the stabilization of SET7/9 *in cellulo*. **c** FTS analysis to evaluate the stability of recombinant His-tagged SET7/9 protein in the presence or absence of **Set7_1a** (10 μM). **d** ITC titration of recombinant SET7/9 protein (500 μM) into **Set7_1a** (50 μM). **e** BLI sensorgrams of the interaction between **Set7_1a** and recombinant His-tagged SET7/9 protein. **f** Covalent binding of Hcy to **Set7_1a** was confirmed by mass spectrometry. The main adduct formed at 721.2821 (MS positive, apo-**Set7_1a** + Hcy). Apo = Apomictic. **g** The effect of **Set7_1a** with or without Hcy on the SAM-SET7/9 interaction in vitro was estimated using a fluorescence polarization assay. **h** The relative levels of SET7/9 in hyperglycemia-induced HUVECs after Ctr and SET7/9 siRNA treatment. **i** Effect of the **Set7_1a complex** on HIF-1α and SET7/9 levels in hyperglycemia-induced HUVECs with or without SET7/9 knockdown. ^*^P < 0.05, ^**^P < 0.01 vs. the Ctr group.

Target engagement, which describes the capability of a drug to bind to the target protein in vivo, is an important measure of drug efficiency. The cellular thermal shift assay (CETSA) evaluates the thermal stabilization of proteins upon compound binding, which could indicate the target engagement of the candidate compounds *in cellulo*^43^. In this study, the SET7/9 engagement of the most potent inhibitors was evaluated using the CETSA. After incubation in high glucose medium under hypoxia, human umbilical vein endothelial cell (HUVEC) lysates were collected and treated with **Set7_1a** (30 μM) for 30 min. Aliquots were heated to set temperatures, centrifuged to isolate the soluble protein fraction from the cell debris, and then analyzed by immunoblotting (IB) with an anti-SET7/9 antibody. A positive shift of approximately 5 °C in the melting curve of SET7/9, but not β-actin, was observed, indicating stabilization of SET7/9 by the complex **Set7_1a** in cell lysates (Figs. 2a, b). A fluorescence-based thermal shift (FTS) assay was also performed with recombinant SET7/9 in the presence of the complex **Set7_1a** to confirm whether the inhibitors directly bound to the isolated protein. In the presence of 10 μM **Set7_1a**, the *T*_m_ of SET7/9 was increased by approximately 5.7 °C (Fig. 2c), which further suggests that **Set7_1a** directly engages and stabilizes SET7/9. The interaction between **Set7_1a** and SET7/9 was characterized by isothermal titration calorimetry (ITC), which demonstrated a *K*_d_ value of 1.06 ± 0.53 μM between **Set7_1a** and recombinant SET7/9 protein with a 1:1 stoichiometry (N = 0.85), and the binding between **Set7_1a** and recombinant SET7/9 was driven by both enthalpic and entropic factors (Δ*H* = −7.19 kcal/mol, −*T*Δ*S* = −0.964 kcal/mol, Δ*G* = −8.15 kcal/mol) (Fig. 2d and Supplementary Fig. 3). Moreover, biolayer interferometry (BLI) revealed *K*_d_ values of 1.40 ± 0.17 μM (steady-state fit) and 1.54 ± 0.03 μM (kinetic fit) (Fig. 2e).

To further uncover the binding mechanism, we examined the UV/Vis absorption of the complex **Set7_1a** in DMSO and ACN solution. The results suggested that **Set7_1a** could exchange its ACN ligands for DMSO from the solvent (Supplementary Fig. 4a). Similar solvent exchange reactions have been reported for other complexes, such as complex **Set7_1a**^40^, NAMI-A, and KP1019^44^. We also detected the stability of **Set7_1a** in DMSO solution by UV/Vis absorption spectroscopy (Supplementary Fig. 4b). The lack of significant shifts in the absorbance spectrum suggested that **Set7_1a** was stable in DMSO solution at 298 K for at least 48 h. Moreover, the absorbance of **Set7_1a** was not significantly changed after coincubation with SAM or SAH, suggesting that **Set7_1a** does not interact directly with SAM or SAH to inhibit SET7/9 activity (Supplementary Fig. 4c). Moreover, in the presence of 20 common amino acids, the complex **Set7_1a** displayed a selective response toward cysteine (Cys) with diminished luminescence at *I*_max_ = 625 nm (Supplementary Fig. 5a). However, a previous study showed that Cys blood levels in diabetes are reduced due to elevated glucagon concentrations, which leads to increased uptake of glutamine and glycine by the liver^45,46^. Additionally, SET7/9 methyltransferase does not have the Cys-rich regions in the postSET domain. These observations suggest that **Set7_1a** does not directly bind to Cys or Cys residues to regulate SET7/9 methyltransferase activity. Thus, we also evaluated the interaction of **Set7_1a** with Hcy, which is a homolog of Cys (Supplementary Fig. 5b). Hcy levels are increased in patients with diabetes, especially in those with type 2 diabetes, but also in prediabetic individuals with insulin resistance^47^. Interestingly, the complex **Set7_1a** emitted a comparable diminished luminescence response in the presence of Hcy. To validate the possible action mechanism of **Set7_1a**, we performed HRMS analysis on a mixture of the complex **Set7_1a** and Hcy after 30 min of incubation at room temperature (Fig. 2f). A fragment ion was detected at *m*/*z* = 600.98 (apo-**Set7_1a**), which corresponded to the loss of two can moieties from **Set7_1a**. Moreover, a new peak with *m*/*z* = 736.11 appeared, which matched the theoretical molecular weight of the apo-**Set7_1a**–Hcy complex. The ligand exchange with amino acids has also been previously observed with other metal complexes in the literature^40,48^.

To gauge the specificity of **Set7_1a**, we also measured its potency against PR-SET7 (also known as SET8, SETD8, KMT5A), which is structurally similar to SET7/9^49^. The results showed that **Set7_1a** exhibited no significant effect on PR-SET7 thermal stability in the FTS assay (Supplementary Fig. 6a). Moreover, since **Set7_1a** interacted with Cys amino acids (Fig. 2f and Supplementary Fig. 5a), we also tested its interaction with two conserved Cys-rich proteins, Kelch-like ECH-associated protein 1 (Keap1) and 8-oxoguanine DNA glycosylase 1 (OGG1), which contain 27 and 8 Cys residues^50,51^, respectively. The results showed that **Set7_1a** did not significantly stabilize Keap1 (Supplementary Fig. 6b) or OGG1 (Supplementary Fig. 6c) in the FTS assay. Taken together, these data indicate that **Set7_1a** potently and selectively engages SET7/9.

We next evaluated the ability of Hcy to enhance the ability of **Set7_1a** to disrupt the interaction between the SAM probe and SET7/9 using the in vitro FP assay. As shown in Fig. 2g, preincubation of **Set7_1a** with Hcy further disrupted the interaction between the SAM probe and SET7/9 compared to **Set7_1a** alone. These results indicated that the covalent complex between **Set7_1a** and Hcy is even more effective at engaging the SAM binding pocket of SET7/9 compared to **Set7_1a**.

As the activity of SET7/9 reduces the stability of HIF-1α, we wanted to explore whether **Set7_1a** could stabilize HIF-1α *in cellulo* and investigate whether this stabilization was dependent on SET7/9. SET7/9 knockdown cells were prepared by treating hyperglycemia-induced HUVECs with SET7/9 siRNA. As depicted in Fig. 2h, treatment of the control siRNA cells with **Set7_1a** or sinefungin resulted in significant HIF-1α accumulation. However, the HIF-1α levels were relatively unaffected by **Set7_1a** or sinefungin in the SET7/9 knockdown cells (Fig. 2i). This finding suggests that the accumulation of HIF-1α induced by **Set7_1a** requires the presence of SET7/9 protein in hyperglycemia-induced HUVECs.

We also evaluated the selectivity of **Set7_1a** by evaluating its effect on other negative regulators of HIF-1α. The results showed that **Set7_1a** does not interfere with proteasome activity, oxygen consumption, PHD2, or VHL levels (Supplementary Fig. 7), indicating that SET7/9 is the main target of **Set7_1a** to activate the HIF signaling axis, which suggests that **Set7_1a** may function as a selective HIF-1α stabilizer.

### Set7_1a inhibits SET7/9-mediated HIF-1α lysine methylation at the lysine-32 residue

**Fig. 3:**
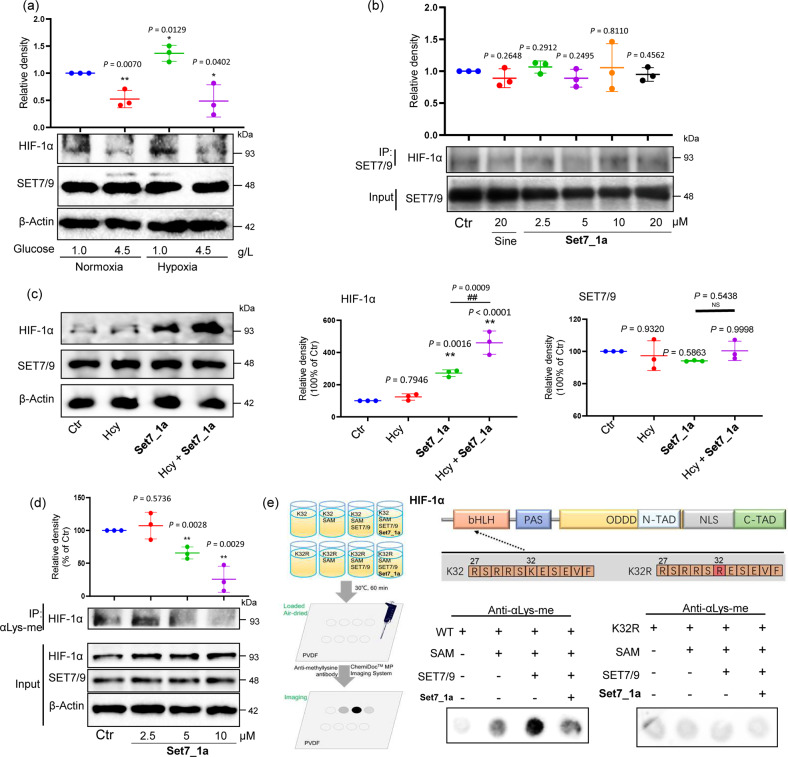
Set7_1a inhibits SET7/9-mediated HIF-1α lysine methylation at the lysine-32 residue. **a** Western blot analysis to evaluate the effect of glucose levels on the stabilization of HIF-1α in HUVECs under normoxic and hypoxic conditions. **b** The effect of **Set7_1a** on the interaction of SET7/9 and HIF-1α *in cellulo* using the co-IP assay. **c** Western blot analysis to evaluate the effect of the **Set7_1a complex** (10 μM) with or without Hcy on HIF-1α and SET7/9 levels in the hyperglycemia-induced HUVECs under hypoxia. Data are expressed as the mean ± SD (*n* = 3 independent experiments), ^*^P < 0.05, ^**^P < 0.01 *vs*. the Ctr group; ^##^*P* < 0.01, ^NS^*P* > 0.05, the **Set7_1a** *vs*. Hcy + **Set7_1a** group. **d** Effect of **Set7_1a** on the HIF-1α methylation level in the hyperglycemia-induced HUVECs. **e** Effect of **Set7_1a** on the methylation of HIF-1α peptide mediated by Set7/9. Dot blot analysis of the synthesized HIF-1α peptide (WT) methylated by Set7/9 in vitro was performed with an anti-methyl lysine antibody. The HIF-1α peptide with lysine to arginine substitution (K32R) was used as a negative control for antibody detection. Protein domains of HIF-1α: bHLH, basic helix-loop-helix; PAS, Per-ARNT-Sim; N-TAD, NH2-terminal transactivation domain; ODDD, oxygen-dependent degradation domain; NLS, nuclear localization signal; C-TAD, COOH-terminal TAD. ^*^P < 0.05, ^**^P < 0.01 *vs*. the Ctr group.

A coimmunoprecipitation (co-IP) assay was performed to examine whether **Set7_1a** disrupted the interaction between HIF-1α and SET7/9 *in cellulo*. First, we demonstrated that hyperglycemia decreased the levels of HIF-1α under both hypoxic and normoxic conditions in HUVECs without affecting the level of SET7/9 (Fig. 3a). High glucose is known to promote SET7/9 nuclear localization in human endothelial cells but does not alter SET7/9 expression^52^. In the co-IP results, **Set7_1a** did not decrease the amount of HIF-1α coprecipitating with SET7/9 in hyperglycemia-induced HUVECs in hypoxia, indicating that it did not affect the interaction of SET7/9 and HIF-1α in the treated cells (Fig. 3b). Interestingly, we found that **Set7_1a** increased HIF-1α levels in a dose-dependent manner (Fig. 2i). Furthermore, cotreatment with Hcy, as described previously^53^, further increased HIF-1α levels in hyperglycemia-induced HUVECs compared to **Set7_1a** alone, demonstrating that Hcy could enhance the potency of **Set7_1a** to induce the stabilization of HIF-1α but not affecting the level of SET7/9 (Fig. 3c and Supplementary Fig. 8).

HIF-1α possesses a consensus sequence that is recognized and methylated by SET7/9 near the lysine-32 site, leading to the ubiquitin-mediated degradation of HIF-1α^54^. To further study the mechanism by which **Set7_1a** prevents HIF-1α degradation, we performed immunoprecipitation with an anti-methyl lysine antibody in hyperglycemia-induced HUVECs treated with **Set7_1a** for 12 h under hypoxia. Afterward, IB analysis was performed with an anti-HIF-1α antibody. The results showed that HIF-1α lysine methylation was significantly decreased in a dose-dependent manner (Fig. 3d). To confirm whether **Set7_1a** could inhibit HIF-1α methylation specifically at the lysine-32 residue, we also conducted an in vitro methylation experiment using purified recombinant SET7/9 enzymes and synthesized HIF-1α wild-type (Supplementary Fig. 9) or K32R (Supplementary Fig. 10) peptides as substrates. **Set7_1a** significantly inhibited the lysine methylation level of HIF-1α wild-type (WT) peptides as shown by IB with an anti-methyl lysine antibody, while it had no effect on HIF-1α K32R peptides (Fig. 3e). These results suggested that **Set7_1a** could significantly inhibit SET7/9-mediated HIF-1α lysine methylation at the lysine-32 residue. This observation supports the hypothesis that **Set7_1a** (or apo-**Set7_1a**–Hcy) disrupts the interaction between SET7/9 and SAM/SAH rather than disrupting the interaction between SET7/9 and HIF-1α.

### Set7_1a upregulates HIF-1α target protein expression and promotes angiogenesis *in cellulo* and in vivo

**Fig. 4:**
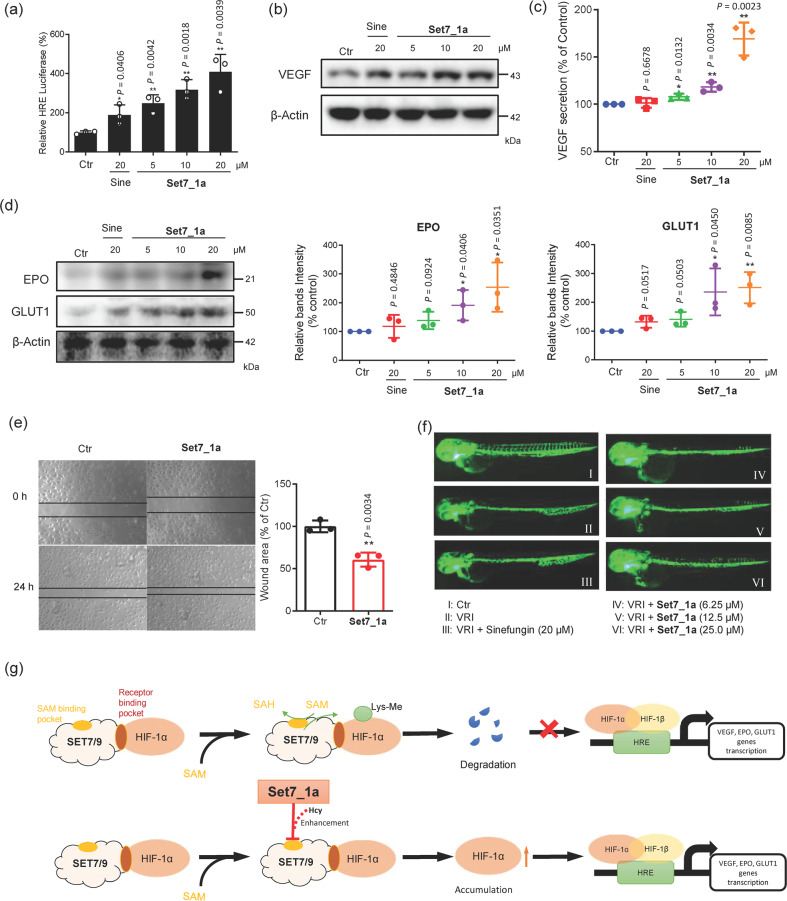
Effect of Set7_1a on angiogenesis. **a** Effect of the **Set7_1a** complex on HRE luciferase activity in hyperglycemia-induced HUVECs under hypoxic conditions. **b** Effect of **Set7_1a** on the levels of VEGF protein in the hyperglycemia-induced HUVECs under hypoxic conditions. **c** The relative VEGF levels in the hyperglycemia-induced HUVECs under hypoxic conditions. VEGF concentration was determined by ELISAs. **d** Effect of the **Set7_1a complex** on the levels of EPO and GLUT1 proteins in the hyperglycemia-induced HUVECs under hypoxic conditions. **e** The effect of the **Set7_1a** complex on HUVEC migration was assessed using an in vitro wound-healing assay. Ninety percent confluent monolayers of HUVECs were scratch wounded and then incubated with **Set7_1a** (5 μM) for 24 h. **f** Microangiography of zebrafish embryo blood vessels. VRI was introduced to inhibit the angiogenesis of the zebrafish embryos. **g** Schematic model of the effect of **Set7_1a** on gene transcription. ^*^P < 0.05, ^**^P < 0.01 *vs*. the Ctr group.

HIF-1α translocates into the nucleus, thus binding to HRE to promote a hypoxic response. In this study, the ability of **Set7_1a** to enhance the transcription of HIF-1α genes *in cellulo* was evaluated using a dual luciferase reporter assay. **Set7_1a** increased HRE-driven luciferase intensity (after normalization to Renilla luciferase activity as an internal control) in a dose-dependent manner (Fig. 4a). This result suggests that by inhibiting the methylation activity of SET7/9 *in cellulo*, HIF-1α is allowed to accumulate and translocate into the nucleus to activate gene expression.

HIF-1α activity drives the expression of several target genes involved in angiogenesis, including *VEGF*, *EPO*, and *GLUT1*^16,55^. Therefore, the effect of **Set7_1a** on VEGF secretion from hyperglycemia-induced HUVECs under hypoxia was also investigated. **Set7_1a** treatment for 12 h in high-glucose medium under hypoxia increased VEGF secretion from HUVECs in a dose-dependent manner, as determined by Western blotting (Fig. 4b), which was also verified by ELISAs (Fig. 4c). Under similar conditions, the expression levels of GLUT1 and EPO were also increased by **Set7_1a** (Fig. 4d).

HIF-1α plays a key role in enhancing the hypoxic induction of epithelial cell migration during wound closure^56^. To evaluate the effect of **Set7_1a** on wound closure, we performed the scratch assay, a widely used experiment to assess wound healing *in cellulo*. HUVECs were grown to confluency as monolayers on plastic and scratched with a 200 μL pipette tip. **Set7_1a** (5 μM) treatment promoted the wound repair of HUVECs (Fig. 4e), as indicated by a significant decrease in the wound area versus the control. Additionally, although **Set7_1a** exhibited moderate cytotoxicity toward HUVECs, with an IC_50_ value of 29.5 μM under hypoxic conditions after 48 h (Supplementary Fig. 11), the concentrations of **Set7_1a** required to induce beneficial biological effects were much lower than its IC_50_ value. Moreover, the complex **Set7_1a**-stimulated migration of HUVECs was significantly enhanced in the presence of Hcy (100 μM) after 24 h (Supplementary Fig. 12). This result demonstrates that Hcy is a potential enhancer in **Set7_1a**-mediated functions in living HUVECs and supports the hypothesis that **Set7_1a** can exert enhanced beneficial biological effects at lower concentrations than its in vitro IC_50_ value.

HIF-1α expression is upregulated in hypoxia and is often associated with the induction of angiogenesis^57^. Given the promising ability of **Set7_1a** to promote HIF-1α target genes, we hypothesized that **Set7_1a** could potentially promote angiogenesis in vivo. Zebrafish is a commonly used angiogenesis model because it is fast-growing and optically transparent. In this study, zebrafish embryos at 20 hours post-fertilization (hpf) were pretreated with a known angiogenesis inhibitor, VEGFR tyrosine kinase inhibitor II (VRI), for 4 h and then incubated with **Set7_1a** or sinefungin for an additional 24 h. As expected, in the absence of **Set7_1a**, VRI strongly suppressed angiogenesis, with almost no blood vessel formation observed in the zebrafish trunk (Fig. 4f). Excitingly, **Set7_1a** (6.25 to 25 μM) could attenuate VRI-induced inhibition of angiogenesis in vivo in a dose-dependent manner, as revealed through an increase in blood vessel formation. Moreover, 6.25 μM of **Set7_1a** was more effective at promoting angiogenesis than 20 μM sinefungin. This result indicates that **Set7_1a** has the potential to be developed as a therapeutic agent to promote angiogenesis or wound healing. We hypothesize that the enhancement of angiogenesis or wound healing can be attributed, at least in part, to the ability of **Set7_1a** to inhibit the catalytic activity of SET7/9 and enhance HIF-1α stability, thus leading to an accumulation of HIF-1α-driven proangiogenic products (Fig. 4g).

### Set7_1a accelerates wound healing in mimic type 2 diabetic mice

**Fig. 5:**
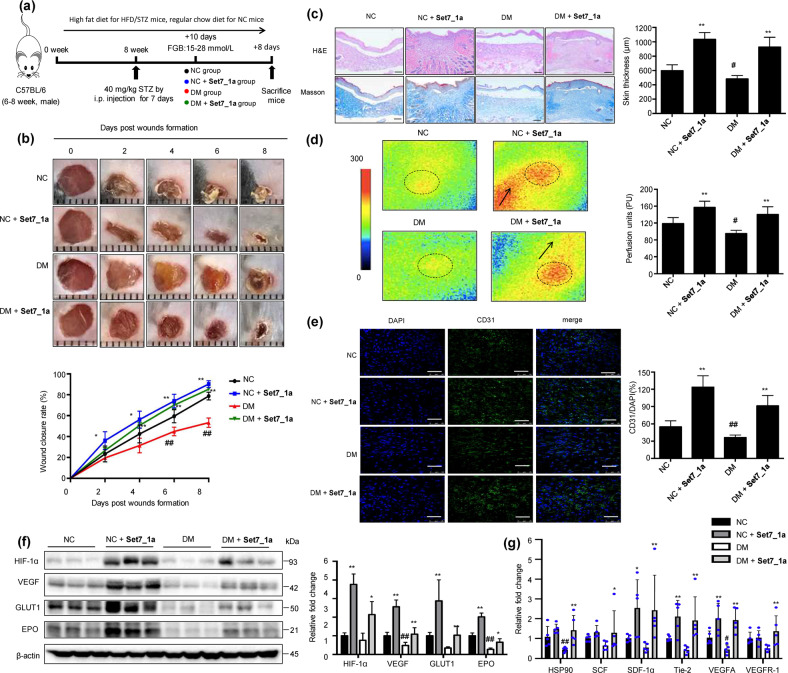
Set7_1a accelerates wound closure in mimic type 2 diabetic mice. **a** Timeline for the mouse animal study. **b** Representative images of wound (up) and wound closure rates (down). **c** H&E staining and Masson’s trichrome staining of dorsal skin sections (left) and skin thickness (right). Scale bar = 200 μm. **d** Representative images for each group (left) and baseline perfusion on the back skin of mice (right). **e** DAPI and CD31 staining in the wound bed. Scale bar = 50 μm. **f** IB (left) and quantitative (right) analyses of HIF-1α target genes in wound tissue. **g** The mRNA levels of proangiogenic genes involved in wound healing. Data are expressed as the mean ± SD (*n* = 5–8 mice). ^#^*P* < 0.05, ^##^*P* < 0.01 DM *vs*. NC, *P < 0.05, **P < 0.01 the complex **Set7_1a** *vs*. vehicle.

Finally, the role of **Set7_1a** in wound healing was evaluated in normal and type 2 mimic diabetic mice. The mice were fed a high-fat diet for 8 weeks, followed by low-dose STZ (40 mg/kg) injection daily for 1 week (Fig. 5a). Three days after the last STZ injection, the mice with 15 to 28 mmol/L fasting blood glucose levels were deemed mimic type 2 diabetic mice (DM) and recruited for wound healing experiments (Supplementary Fig. 13a). Vehicle (PEG 400:distilled water = 6:4, *v*/*v*) and **Set7_1a** (50 mg/kg in PEG 400 solution) were then intraperitoneally (i.p.) injected into the vehicle and **Set7_1a** groups, respectively, every other day for 8 days. Inductively-coupled plasma mass spectrometry (ICP-MS) experiments demonstrated the presence of iridium in skin and plasma specimens of treated mice, confirming the delivery of **Set7_1a** (Supplementary Fig. 14). **Set7_1a** treatment did not induce obvious adverse reactions or body weight changes over 8 days in either normal control (NC) or DM mice (Supplementary Fig. 13b). For analysis of wound healing, two full-thickness skin lesions were excised in the interscapular area of each animal, the wounds were monitored every other day, and wound closure rates were calculated for each group. Encouragingly, treatment with **Set7_1a** dramatically accelerated wound healing in both the NC and DM mice (Fig. 5b). In the NC groups, the **Set7_1a**-treated mice showed 36%, 56% and 74% closure at 2, 4 and 6 days postinjury and almost completed wound closure by Day 8 postinjury, whereas the vehicle-treated mice exhibited 23%, 42%, 59% and 78% closure after 2, 4, 6 and 8 days postinjury, respectively (Fig. 5b). In the DM groups, the wound closure rates in the **Set7_1a**-treated mice were 26%, 50%, 70% and 85% at 2, 4, 6 and 8 days postinjury, respectively, and the wound closure rates in the vehicle control mice were nearly 19%, 31%, 44% and 53% at 2, 4, 6 and 8 days postinjury, respectively (Fig. 5b). Taken together, these results indicated that **Set7_1a** accelerates wound healing in both normal and mimic type 2 diabetic mice.

H&E and Masson’s trichrome staining were further employed to assess the epithelial thickness of regenerated skin. The results showed that the dorsal skin thickness was greater in the **Set7_1a**-treated mice than in the vehicle control mice in both the NC and DM groups (Fig. 5c). Masson’s trichrome staining further showed increased collagen deposition in the **Set7_1a complex**-treated mice compared to the vehicle controls (Fig. 5c). Tissue angiogenesis plays a key role in wound healing. The skin perfusion pressure experiments revealed that **Set7_1a** enhanced the skin blood flow rate in both the NC and DM mice (Fig. 5d). Immunostaining for CD31, a specific marker of vascular vessels, further indicated that **Set7_1a** could significantly increase microvessel density in wounding areas in mice from both the NC and DM groups (Fig. 5e). The expression levels of HIF-1α, VEGF, EPO, and GLUT1 were significantly increased in the wound tissue from the **Set7_1a**-treated DM mice compared with those from the vehicle control DM mice (Fig. 5f). Moreover, HIF-1α products that are required for wound healing cell motility (HSP-90), angiogenesis (VEGF-R), and recruitment of CAG (Tie-2, SCF, and SDF-1) were also increased in the wound tissue from the **Set7_1a** treated DM mice (Fig. 5g and Supplementary Table 1). Taken together, **Set7_1a** accelerates wound healing in mimic type 2 diabetic mice by activating HIF-1α signaling and downstream proangiogenic factors.

## Discussion

Patients with diabetes commonly have impaired wound healing that does not progress through the healing process in a timely manner, creating major challenges to healthcare systems worldwide^58^. Recent research has shown that dysfunctional cellular reactions to hypoxic conditions drive impaired wound healing in diabetes. HIF-1α is an important regulator of tissue repair; however, hyperglycemia destabilizes the HIF-1α protein in hypoxia^59^. Biopsies from individuals with DFU exhibit decreased HIF-1α levels compared to those from individuals with chronic venous ulcers^60^. As a protein lysine methyltransferase, SET7/9 plays an important role in controlling the HIF-1α level. HIF-1α methylation by SET7/9 leads to protein degradation of HIF-1α, thus diminishing the transcription of HIF-1α target genes, including those responsible for angiogenesis and wound healing. Recent studies showed that sustained vascular gene expression of methyltransferase SET7/9 was enhanced in diabetes as a response to elevated glucose in vascular endothelial cells^24^. Interestingly, enhanced levels of SET7/9 recruitment were uncovered in macrophages from diabetic mice, and SET7/9-induced epigenetic transitions have been linked to vascular dysfunction in type 2 diabetic patients^61^. In this context, HIF-1α stabilization by inhibiting SET7/9 function and antagonizing HIF-1α methylation thus becomes a possible strategy to promote angiogenesis to enhance wound healing in diabetes.

**Fig. 6:**
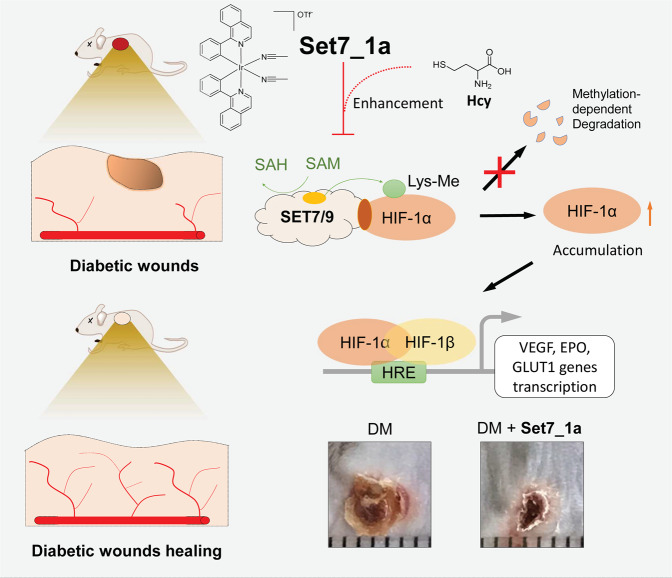
Summary diagram. This work reports the discovery of a homocysteine-targeting complex **Set7_1a** as a SET7/9 methyltransferase inhibitor that regulates the methylation-dependent degradation of HIF-1α, an important target protein for improving diabetic wound healing. **Set7_1a** could improve wound healing in mimic type 2 diabetic mice by activating HIF-1α signaling, opening an avenue for treating diabetic wounds via SET7/9 inhibition using homocysteine-targeting compounds.

Here, we discovered that the Ir(III) complex **Set7_1a** bearing ACN ligands is a potent inhibitor of SET7/9 activity. This complex strongly inhibits SET7/9 activity, especially after preincubation with Hcy, an elevated factor in diabetes. Cell-based and cell-free assays suggest that **Set7_1a** (or apo-**Set7_1a**–Hcy) engages SET7/9 to disrupt its interaction with SAM. Moreover, the **Set7_1a** complex effectively induced HIF-1α accumulation and upregulated HIF-1α target protein expression involved in angiogenesis. Importantly, **Set7_1a** was able to improve wound healing in diabetic mice by activating HIF-1α signaling and downstream proangiogenic factors (Fig. 6). This study demonstrates a Hcy-targeting iridium compound as a SET7/9 inhibitor for accelerating diabetic wound healing and, more importantly, opens a novel avenue for the treatment of diabetic wounds by the inhibition of SET7/9 lysine methyltransferase activity.

Elevated levels of Hcy, a nonessential sulfur-containing amino acid, have been reported as a risk factor in diabetes^62,63^. Thus, an effective treatment for lowering elevated Hcy in chronic wound patients may promote the return of normal healing processes. Interestingly, our study demonstrates a treatment strategy using the selective SET7/9 inhibitor **Set7_1a**, which could interact with Hcy via ligand exchange, enhance HIF-1α stabilization of HUVECs in the presence of Hcy, and promote the expression of angiogenic proteins, such as VEGF, GLUT1 and EPO, in hypoxia and hyperglycemia. Hence, this complex may have the potential to correct elevated Hcy levels via covalent binding in a patient with impaired wound healing.

HIF-1α target genes (e.g., *VEGF*, *EPO*, and *GLUT1*) mediated by **Set7_1a** are factors that are responsible for angiogenesis and diabetic wound healing. For example, VEGF, as an endothelial cell mitogen and inducer of vascular permeability, has been identified as a crucial factor for the altered production of new blood vessels, which is one of the compromised phases in diabetic wound healing^64,65^. As a hematopoietic factor regulating the proliferation and differentiation of erythroid precursor cells, EPO was successfully used to improve diabetes-impaired wound healing by stimulating macrophage function and activity, which is responsible for the production of cytokines and growth factors that are essential for the healing cascade^66^. Apart from VEGF and EPO, GLUT1, as a mediator of a limiting step in glucose metabolism, enhances the wound repair process by regulating glycolysis and ROS production^67,68^. Moreover, other pathways independent of **Set7_1a**-mediated activation of these target genes, such as the Wnt/β-catenin, Notch and JAK/STAT signaling pathways, may be involved in promoting the wound healing process.

Notably, our previously published VHL-HIF-1α interaction blocker also showed HIF-1α stabilization and wound healing effects similar to the SET7/9 inhibitor **Set7_1a** reported in this study. However, **Set7_1a** could promote HIF-1α stability by disrupting the interaction between SET7/9 and SAM/SAH and blocking HIF-1α lysine methylation rather than disrupting the interaction between SET7/9 and HIF-1α, which is different from the mechanism of the VHL-HIF-1α interaction blocker. These results indicate that the combination of complexes **Set7_1a** and **1a** may be a more potent strategy to induce the stabilization of HIF-1α by simultaneously blocking methylation-dependent degradation (HIF-1α lysine methylation) and hydroxylation-dependent degradation (VHL-HIF-1α interaction), which could open a new research direction for treating diabetic wound healing in the future.

## Supplementary information

SUPPLEMENTAL MATERIAL
