# Supplementary material for: Homocysteine-targeting compounds as a new treatment strategy for diabetic wounds via inhibition of the histone methyltransferase SET7/9
Source: Exp Mol Med. 2022 Jul 20;54(7):988–98. doi: 10.1038/s12276-022-00804-1 (PMC9356058; doi:10.1038/s12276-022-00804-1)
Supplement: Supplementary file 1 — SUPPLEMENTAL MATERIAL [file 12276_2022_804_MOESM1_ESM.pdf]

Supplementary Information

# Homocysteine-targeting compounds as a new treatment strategy for diabetic wounds via inhibition of the histone methyltransferase SET7/9

Guodong Li,[1,4,6] Dan Li,[1,6] Chun Wu,[2,6] Shengnan Li,[1] Feng Chen,[1] Peng Li,[1] Chung-Nga Ko,[2] Wanhe Wang,[2,5] Simon Ming-Yuen Lee,[1] Ligen Lin,[1,*] Dik-Lung Ma[2,*] and Chung-Hang Leung[1,3,4,*]

[1] State Key Laboratory of Quality Research in Chinese Medicine, Institute of Chinese Medical Sciences, University of Macau, Macao, China. [*]E-mail: duncanleung@um.edu.mo; LigenL@um.edu.mo

[2] Department of Chemistry, Hong Kong Baptist University, Kowloon Tong, Hong Kong, China. [*]E-mail: edmondma@hkbu.edu.hk

[3] Department of Biomedical Sciences, Faculty of Health Sciences, University of Macau, Macao, China.

[4] Zhuhai UM Science and Technology Research Institute, Zhuhai 519031, China

[5] Institute of Medical Research, Northwestern Polytechnical University, Xi'an, Shaanxi, China

[6] These authors contributed equally: Guodong Li, Dan Li, Chun Wu.

Methods

*1.1. Synthesis of rhodium(III) and Iridium(III) complexes*

Preparation of the precursor complexes [$M_2$(C^N)$_4$$Cl_2$]. Cyclometalated dichloro-bridged dimers of the general formula [$M_2$(C^N)$_4$$Cl_2$], where M = Ir(III)/Rh(III), were synthesized according to a literature method. Specifically, a solution of rhodium(III) chloride or iridium(III) chloride (1 eq.) and corresponding C^N ligands (2.1 eq.) in a mixture of methoxyethanol:water (v/v, 3:1) was heated under reflux overnight under a nitrogen atmosphere. The reaction mixture was cooled to room temperature. The solid was collected by filtration, washed with additional portions of water (2 × 100 mL) and diethyl ether (2 × 50 mL) and dried to yield the corresponding precursor complexes.

General synthesis of [M(C^N)$_2$(ACN)$_2$]OTf complexes. These complexes were synthesized according to a literature method. In brief, [M(C^N)$_2$(ACN)$_2$]OTf was mixed with 2.0 equivalents of silver triflate in 25 mL acetonitrile and stirred at room temperature under a nitrogen atmosphere for 15 h. The mixture was filtered and washed with two portions of ether (2 × 30 mL) to yield titled product.

The synthesis of [M(C^N)$_2$(N^N)]$PF_6$ complexes. A suspension of [$Rh_2$(C^N)$_4$$Cl_2$] or [$Ir_2$(C^N)$_4$$Cl_2$] (0.2 mmol) and corresponding N^N ligands (0.42 mmol) in a mixture of dichloromethane:methanol (1:1, 20 mL) was refluxed overnight under a nitrogen atmosphere. The resulting solution was then allowed to cool to room temperature, and filtered to remove unreacted cyclometallated dimer. To the filtrate, an aqueous solution of excess ammonium hexafluorophosphate was added and the filtrate was reduced in volume by rotary evaoration until precipitation of the crude product occurred. The precipiate was then filtered and washed with several portions of water (2 × 50 mL) followed by diethyl ether (2 × 50 mL). The product was recrystallized by acetone:diethyl ether vapor diffusion to yield the titled compounds.

**Complex Set7_1**: Reported[1]

**Complex Set7_2**: Reported[1]

**Complex Set7_3**: Reported[2]

**Complex Set7_4**: Reported[3]

**Complex Set7_5**: Reported[2]

**Complex Set7_6**: Reported[4]

**Complex Set7_7**: Reported[1]

**Complex Set7_8**: Reported[5]

**Complex Set7_9**: Reported[5]

**Complex Set7_10**: Reported[6]

**Complex Set7_11**: Reported[2]

**Complex Set7_12**: Reported[1]

**Complex Set7_13**: Reported[7]

**Complex Set7_14**: Reported[1]

**Complex Set7_15**: Reported[2]

**Complex Set7_16:** Reported[5]

**Complex Set7_17**: Reported[6]

**Complex Set7_18**: Reported[5]

**Complex Set7_1a**: Reported[1]

**Complex Set7_1b**: Reported[1]

**Complex Set7_1c**: Reported[1]

**Complex Set7_1d**: Reported[1]

**Complex Set7_1e**: Reported[1]

**Complex Set7_1f**: $^{1}$H NMR (400 MHz, Acetonitrile-$d_3$) δ 9.03 (d, *J* = 8.7 Hz, 2H), 8.98 (d, *J* = 6.3 Hz, 2H), 8.33 (d, *J* = 8.0 Hz, 2H), 8.23 (d, *J* = 8.2 Hz, 2H), 7.98 (dd, *J* = 13.8, 6.6 Hz, 4H), 7.90 (t, *J* = 7.7 Hz, 2H), 7.08 (t, *J* = 7.6 Hz, 2H), 6.81 (t, *J* = 7.5 Hz, 2H), 6.13 (d, *J* = 7.7 Hz, 2H), 1.98 (d, *J* = 2.2 Hz, 6H). $^{13}$C NMR (100 MHz, Acetonitrile-$d_3$) δ 165.51, 165.18, 164.63, 145.48, 141.79, 137.36, 132.00, 131.59, 129.52, 128.79, 126.09, 122.95, 121.24. MALDI-TOF-HRMS: Calcd. for $C_{34}H_{26}RhN_4[M\text{–}2ACN\text{-}CF_3SO_3]^+$: 511.3987 Found: 511.4302.

*1.2. Materials and cell lines*

Dulbecco's Modified Eagle's Medium (DMEM) and Fetal bovine serum (FBS) were available from Gibco BRL. Human embryonic kidney HEK293 cells (CRL-1573) and Human Umbilical Vein Endothelial Cells (HUVEC)  (CRL-1730) were obtained from ATCC and maintained in Dulbecco's modified Eagle's medium (DMEM, Gibco, USA) containing 10% fetal bovine serum (FBS) at 37 °C and in the presence of 5% $CO_2$. Luciferase reporter assay system was purchased from Promega (Madison, WI, USA). β-actin antibody (4967L) was purchased from Cell Signaling Technology. EPO antibody (ab129452) was purchased from Abcam. All other reagents and chemicals were obtained from commercial sources and used as received. All animal experiments were approved by the Animal Ethical and Welfare Committee of University of Macau (No. ICMS-AEC-2014-06). All procedures involved in the animal experiments were carried out in accordance with the approved guidelines and regulations.

*1.3. Mass spectrometry analysis*

Hcy was incubated with complex **Set7_1a** in Tris–HCl buffer (10 mM Tris–HCl, pH = 7.5, 10 mM KCl) 30 min at room temperature, then was directly injected into an MSQ Plus Single Quad Detector (Thermo Scientific MSQ Plus single quadrupole mass spectrometer, Thermo Scientific, USA) at a flow rate of 5 μL $min^{-1}$. Data were analyzed by the software Data Analysis.

*1.4. Amino acids selectivity assay*

The selectivity of complex **Set7_1a** (10 μM) was tested over possible biologically relevant common amino acids (100 μM), including Cys, Hcy, Glu, Arg, Asp, Tyr, Val, Trp, Pro, Ala, Met, Lys, Gly, His, Phe, Leu, Asn, Ile, Ser, Thr and Gln. A stock solution of complex **Set7_1a** (0.5 mM) was prepared in dimethyl sulfoxide (DMSO) for further use. The complex was diluted in Tris–HCl buffer (10 mM Tris–HCl, pH = 7.5, 10 mM KCl) to a final concentration of 20 μM for 1.5 mL. After that, various amino acids were added into each eppendorf tube with a final concentration of 100 μM. The emission spectra were recorded in a cuvette at 25 °C on a PTI QM-1 spectrofluorometer (Photo Technology International, Birmingham, NJ). The intensity of the emission peak of complex **Set7_1a** in the presence or absence of various amino acids at 597 nm were recorded to obtain the bar chart.

*1.5. Cell viability assay*

The cytotoxicity of the compounds towards normal human cells will be measured by an MTT assay. Hyperglycemia induced human umbilical vein endothelial cells HUVEC will be seeded at a density of 5,000 cells per well in 96-well plates and incubated for 12 h under hypoxia. Compounds dissolved in DMSO will be added to cells followed by incubation for 48 h. Then 10 μL of 5 mg/mL MTT (3-(4,5-dimethylthiazol-2-yl)-2,5-diphenyltetrazolium bromide) reagent will be added to each well. After 4 h incubation in the dark, 100 μL of DMSO will be added to each well, and the intensity of absorbance will be determined by a SpectraMax M5 microplate reader at a wavelength of 570 nm.

*1.6. Western blotting*

The transfected cells from BiFC assay will be harvested to obtain whole-cell extracts by the addition of one volume of 250 mM Tris–HCl (pH 6.8), 20% glycerol, 2% sodium dodecyl sulfate (SDS), 5% 2- mercaptoethanol, and 0.2% bromophenol blue to cells in one volume of PBS followed by boiling for 5 min. Samples will resolved on sodium dodecyl sulfatepolyacrylamide gel electrophoresis (SDS/PAGE) gels and transferred to polyvinylidene fluoride (PVDF) membranes. Blots will be probed with antibodies to SET7/9, HIF-1α, GAPDH (cell signaling). After incubation with secondary antibodies (Santa Cruz Biotechnology), blots will be developed with ECL reagent (Thermo Fisher Scientific).

*1.7. Cellular thermal shift assay*

Cellular thermal shift assay was performed to monitor the target engagement of complex **Set7_1a** in hyperglycemia-induced HUVEC cell lysates. Briefly, 2 × $10^6$ hyperglycemia-induced HUVEC cells cultured under hypoxia will be lysed and collected. 300 mg of cell lysates will be diluted and divided into aliquots (200 µL) using PBS. Each lysate will be treated with the most promising compound for 30 min at room temperature and will be then divided into 6 aliquots of 50 µL and placed into separate PCR tubes. The PCR tubes will be heated individually at different temperatures ranging from 25 °C to 60 °C for 5 min (Applied Biosystems 7500, Life Technologies). The heated lysates will be then centrifuged for 5 min at 13 000 g and the supernatants will be subjected to SDS-PAGE followed by immunoblotting with SET7, HIF-1α protein antibodies (CST, 1: 1000 dilution).

*1.8. SET7/9 protein expression and purification*

The purification of recombinant proteins His-SET7/9 was carried out as described previously[8]. Briefly, pET28a_ SET7/9 plasmids of the *E. coli* DH5-Alpha was transformed into the expression strain *E. coli* (BL21) (DE3). The *E. coli* (BL21) (DE3) expressed the wild type human recombinant SET7/9 protein (His tagged) were cultured in lysogeny broth (LB) medium with 50 µg/mL kanamycin and grew at 37°C until the OD600 of 0.5-0.8. Then induced with 1 mM isopropyl-β-D-thiogalactopyranoside (IPTG) at 15°C for 24 h. The recombinant *E. coli* cells were harvested by centrifugation at 12000 g for 20 min and then homogenized by sonication in binding buffer (20 mM sodium phosphate, 500 mM NaCl, 5 mM imidazole, pH 7.4) and precleared lysates were applied to His GraviTrap columns (GE Healthcare), following kit protocols for purifications. The protein was assayed by SDS-PAGE and stained by using the coomassie brilliant blue G250 (Beyotime, Shanghai, China). pET28 Set9 wt was a gift from Danny Reinberg (Addgene plasmid # 24082; RRID:Addgene_24082).

*1.9. Fluorescence-based protein thermal shift assay*

The protein thermal shift assay with the purified protein was performed as described previously[9]. Briefly, purified human recombinant SET7/9 protein was appropriately diluted in $ddH_2O$. All assay experiments used 2 µg protein per well and final concentration 10 × Sypro Orange and 10 µM of complex **Set7_1a** up to a total volume of 20 µL. The PCR plates were sealed with optical seal, shaken, and centrifuged after protein and compounds were added. Thermal scanning (35 to 62 °C at 1 °C/min) was performed using a real-time PCR setup (Mx3005P Q-PCR system) and fluorescence intensity was measured after every 1 min.

*1.10. Isothermal titration calorimetry*

ITC experiments were carried in a MicroCal PEAQ-ITC Isothermal Titration Calorimeter (Malvern Panalytical), as previously described with minor modification[10]. Briefly, the complex **Set7_1a** and recombinant SET7/9 protein were dialyzed into the ITC buffer (20 mM Bis-Tris, 150 mM NaCl, 2 mM DTT, 5% DMSO) overnight. The SET7/9 protein (500 μM) was titrated against 50 μM of complex **Set7_1a**, consisted of 19 injections of 2 μL complex **Set7_1a** solution at a rate of 2 sec/μL at 150 s time intervals. An initial injection of ligand (0.4 μL) was made and discarded during data analysis. The experiment was carried out at 25 °C while stirring at 750 rpm. The generated data was analyzed using the Setup MicroCal PEAQ-ITC Analysis Software provided by the manufacturer.

*1.11. Biolayer interferometry*

The binding affinities of inhibitors to recombinant SET7/9 were measured by biolayer interferometry on an OctetRed 96 (Fortebio). Ni-NTA biosensors were loaded with His-tagged SET7/9 in BLI kinetics buffer (PBS buffer containing 0.02% Tween 20 and 0.1% BSA), washed in the same buffer and transferred to wells containing complex **Set7_1a** or HIF-1α peptide at indicated concentrations in the same buffer. The Ni-NTA biosensor tips coated with His-tagged protein complex were dipped in increasing concentrations of complex **Set7_1a** for 300 s and subsequently dissociated in the wells containing buffer for another 300 s. Negative control performed with BLI kinetics buffer against Ni-NTA biosensors was subtracted from the sample response against SET7/9-loaded Ni-NTA biosensors. The equilibrium dissociation constant ($K_d$) value for a 1:1 interaction was calculated from the steady state fit. The $K_d$ and associated standard errors were calculated using Octet analysis software.

*1.12. Dual luciferase reporter assay*

To evaluate the effect of the compounds on HIF-1α-directed transcription, HRE activity will be determined using a dual luciferase assay. Briefly, HEK-293 cells will be transiently transfected with pRL-TK and the HIF-1α HRE-luciferase reporter plasmids for 36 h. Hyperglycemia-induced cells will be treated with compounds (10 μM) under hypoxia for 8 h before measurement. Luciferase activity will be measured using a spectrophotometer (Spectra-max M5, Molecular Devices, USA) and will be integrated over a 10 second period. The results will be standardization with the activity of Renilla luciferase.

*1.13. SET7/9 knockdown assay*

Hyperglycemia-induced HUVEC cells were seeded in plates at about 80% confluence in DMEM for 12 h. LipofectamineTM 3000 reagent and siRNAs were gently mixed in FBS-free DMEM medium. After 15 min incubation at room temperature, 500 μL of siRNA-lipid complex

were directly added to cells in 1.5 mL DMEM culture medium. Then, HUVEC cells were incubated at 37 $^{o}C$ in a $CO_2$ incubator for 48 h before use.

*1.14. Dot blot assay for HIF-1α methylation detection*

The samples were incubated at 30 $^{o}C$ for 60 min in a reaction buffer containing 50 mM Tris-HCl (pH 8.5), 5 mM MgCl2, 4 mM DTT, active SET7/9 protein and 1 µM SAM. HIF-1α K32 peptide or K32R peptide (1 µg) were used as substrates. The total volume of the reaction mixture was adjusted to 10 µL. The samples were subjected to dot blot and conducted to detect methylation with an anti-methyllysine antibody.

*1.15. ELISA assay for VEGF detection*

The levels of the VEGF released into the cell culture medium will be determined using a commercial ELISA kit. Briefly, 50 µL of cell culture medium samples, assay buffer and diluted detect antibody will be pipetted into the VEGF antibody coated microplate 96 wells. The plate will be covered with an adhesive strip and incubated at room temperature for 2 h. After washing with wash buffer six times, 100 µL of diluted streptavidin-HRP solution will be added in each well followed by incubation for 45 min. After washing six times, 100 µL of substrate solution will be added to the wells followed by incubation for 5-30 min. Color development will be stopped by adding 100 µL of stop solution and the intensity of the color will be measured by a spectrophotometer (Spectra-max M5, Molecular Devices, USA). The endogenous VEGF levels may also be detected *via* immunoblotting with anti-VEGF antibody.

*1.16. Measurement of proteasome activity*

The commercial Proteasome Activity Fluorometric Assay Kit (BioVision Incorporated, CA, USA) was used to detect the proteasome activity after complex **Set7_1a** treatment.

*1.17. Measurement of oxygen consumption*

The effect of complex **Set7_1a** towards oxygen consumption was determined using commercial oxygen consumption rate assay kit (Cayman Chemical, MI, USA).

*1.18. HIF-1α methylation assay*

HUVEC cells grown for 48 h in normal (5.5 mM) or high glucose concentrations (30 mM) will be incubated for an additional 24 h in hypoxia (1% O2) in the presence of the most promising compound or DMSO. Proteins will be extracted and analyzed by Western blotting. An anti-methyl HIF-1α antibody will be applied to examine whether HIF-1α methylation occurs after treatment with the active compounds in hyperglycemia-induced cells.

*1.19. Zebrafish experiments*

Transgenic Tg(fli-1:EGFP) zebrafish will be kept separately with a 14 h light/10 h dark cycle under standard conditions. Zebrafish embryos will be generated by natural pair-wise mating (3–

12 months old) and will be raised at 28.5 °C in embryo water. 24 hpf zebrafish embryos will be collected, distributed into a 12-well microplate with 6 fish in each well and co-treated with 300 nM VRI (VEGFR tyrosine kinase inhibitor II) and the most promising compounds for 24 h. Embryos receiving embryo water with 0.1% DMSO served as a vehicle control and will be equivalent to no treatment. All of these experiments will be repeated three times, with 8 embryos per group.

*1.20. Histological analysis and microvessel density assay*

After fixation in 4% paraformaldehyde, the skin samples were embedded in paraffin and sectioned (5 μm). For histological evaluation, sections were deparaffinized and rehydrated, followed by hematoxylin and eosin (H&E) or Masson's trichrome staining. For immunohistochemical staining of CD31, the wound tissue sections were deparaffinized and stained with CD31 antibody (ABclonal, Cambridge, MA, USA). The slides were examined under ×400 magnification to identify the area with the highest vascular density, and five randomly high-power field areas of the highest microvessel density were selected for each section. The average was calculated as the microvessel density of this sample.

*1.21. Skin wound model*

Dorsum was clipped in anesthetized mice, and then two full-thickness wounds were made with a 6-mm biopsy punch on each side of midline. A circular reference was placed alongside to permit correction for the distance between the camera and the animals. Wound areas were measured every other day by taking digital photographs and quantitated using ImageJ (National Institutes of Health). The wound closure rates were calculated as the following formulation: (wound area on day 0 – wound area on day X)/wound area on day 0 × 100%.

*1.22. Quantitative RT-PCR*

Total RNA of wound tissue was extracted using TRIzol Reagent (Invitrogen, Carlsbad, CA, USA). The cDNA was synthesized from 1 μg RNA using the SuperScript III First-Strand Synthesis System (Invitrogen). Quantitative RT-PCR was performed on a Step-One plus real-time PCR System (Thermo Fisher Scientific, Waltham, MA, USA) using the SYBR green PCR Master Mix, according to the manufacturer's recommended procedures. The primers were listed in Supplementary Table 1.

References

1 Zhong, H.-J. *et al.* A Rhodium (III) complex as an inhibitor of neural precursor cell expressed, developmentally down-regulated 8-activating enzyme with in vivo activity against inflammatory bowel disease. *J. Med. Chem.* **60**, 497-503 (2016).

2 Zhong, H.-J. *et al.* An iridium (III)-based irreversible protein–protein interaction inhibitor of BRD4 as a potent anticancer agent. *Chem. Sci.* **6**, 5400-5408 (2015).

3 Zhou, Y. *et al.* Protein staining agents from cationic and neutral luminescent iridium (III) complexes. *Chem. Eur. J.* **22**, 16796-16800 (2016).

4 Ma, D. L. *et al.* Antagonizing STAT3 dimerization with a rhodium (III) complex. *Angew. Chem. Int. Ed.* **53**, 9178-9182 (2014).

5 Liu, L.-J. *et al.* Inhibition of the Ras/Raf interaction and repression of renal cancer xenografts in vivo by an enantiomeric iridium (III) metal-based compound. *Chem. Sci.* **8**, 4756-4763 (2017).

6 Wang, J.-Q., Hou, X.-J., Bo, H.-B. & Chen, Q.-z. A cyclometalated iridium (III) complex that induces apoptosis in cisplatin-resistant cancer cells. *Inorg. Chem. Commun.* **61**, 31-34 (2015).

7 Leung, C.-H. *et al.* A metal-based tumour necrosis factor-alpha converting enzyme inhibitor. *Chem. Commun.* **51**, 3973-3976 (2015).

8 Nishioka, K. *et al.* Set9, a novel histone H3 methyltransferase that facilitates transcription by precluding histone tail modifications required for heterochromatin formation. *Genes Dev.* **16**, 479-489 (2002).

9 Li, G. *et al.* A small molecule HIF-1α stabilizer that accelerates diabetic wound healing. *Nat. Commun.* **12**, 3363 (2021).

10 Horowitz, S., Yesselman, J. D., Al-Hashimi, H. M. & Trievel, R. C. Direct evidence for methyl group coordination by CH··· O hydrogen bonds in SET domain methyltransferases. *J. Biol. Chem.* **286**, 18658-18663 (2011).

Supplementary Tables

**Supplementary Table 1.** Real-time PCR primer sequences using in this paper.

| Genes name | Forward primer | Reverse primer |
|---|---|---|
| 18S | AGCCTGCGGCTTAATTTGAC | CAACTAAGAACGGCCATGCA |
| HSP-90α | GGACCAGGTTGCTAACTCCG | GGTCTTGCCCTCAAATTCCTT |
| VEGFA | CTTGTTCAGAGCGGAGAAAGC | ACATCTGCAAGTACGTTCGTT |
| VEGFR1 | TGGACCCAGATGAAGTTCCC | GCGATTTGCCTAGTTTCAGTCT |
| SDF1α | GAGAGCCACATCGCCAGAG | TTTCGGGTCAATGCACACTTG |
| SCF | CCTTAGGAATGACAGCAGTAGC | AGCCAATTACAAGCGAAATGAG |
| Tie-2 | GTGTAGTGGACCAGAAGG | CTTGAGAGCAGAGGCATC |

Supplementary Figures

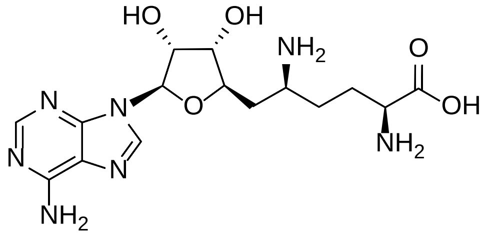


**Supplementary Fig. 1.** Chemical structure of sinefungin (Sine).

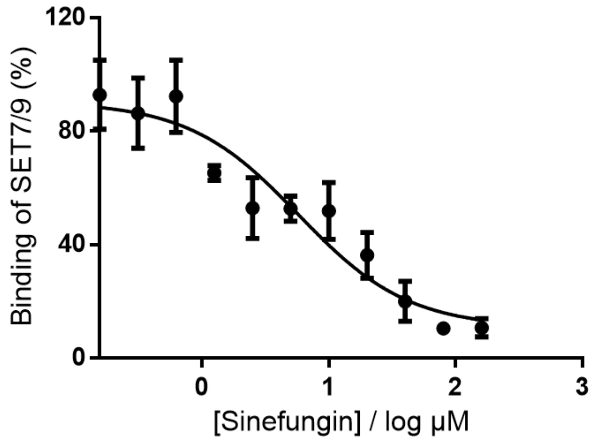


**Supplementary Fig. 2.** Dose-dependent effect of positive control sinefungin with SET7/9 *in vitro* was estimated using an FP assay. Sinefungin displays a SAM site-binding probe in SET7/9 with an $IC_{50}$ of about 6.17 μM. Error bars represent the standard deviations of the results obtained from three independent experiments.

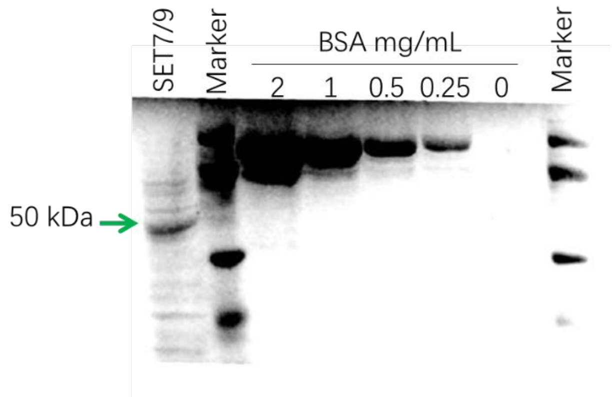


**Supplementary Fig. 3.** Bacterially expressed recombinant human wild type SET7/9 protein (Red arrow) was resolved on 10% SDS-PAGE and visualized by G250 staining. BSA: Bovine serum albumin.

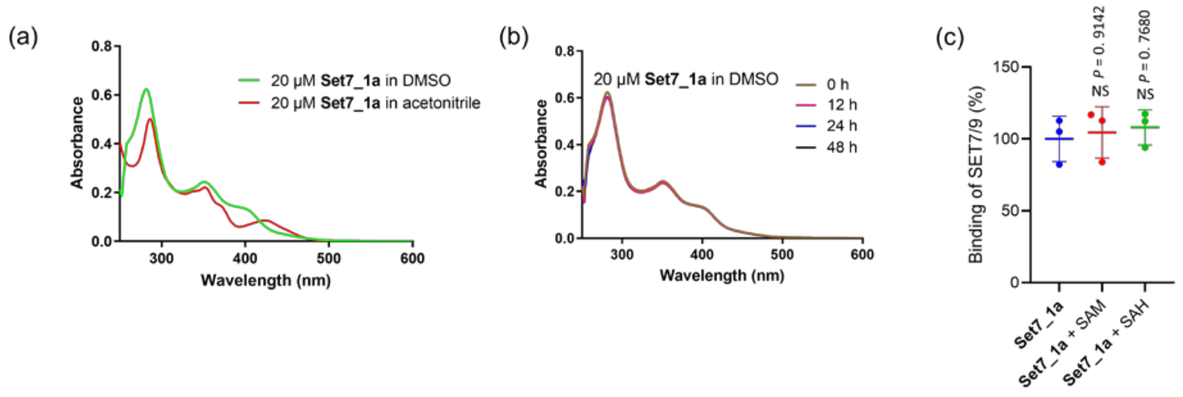


**Supplementary Fig. 4.** (a) UV/Vis absorption of complex **Set7_1a** in DMSO and acetonitrile. (b) UV/Vis absorption of complex **Set7_1a** in DMSO at 298 K over 2 days. (c) Relative emission intensity of complex **Set7_1a** (2 µM) at maximum emission wavelength in Tris–HCl buffer (10 mM Tris–HCl, pH = 7.5, 10 mM KCl) with or without SAM or SAH (4 µM) after incubation at room temperature for 30 min. NS: not significant.

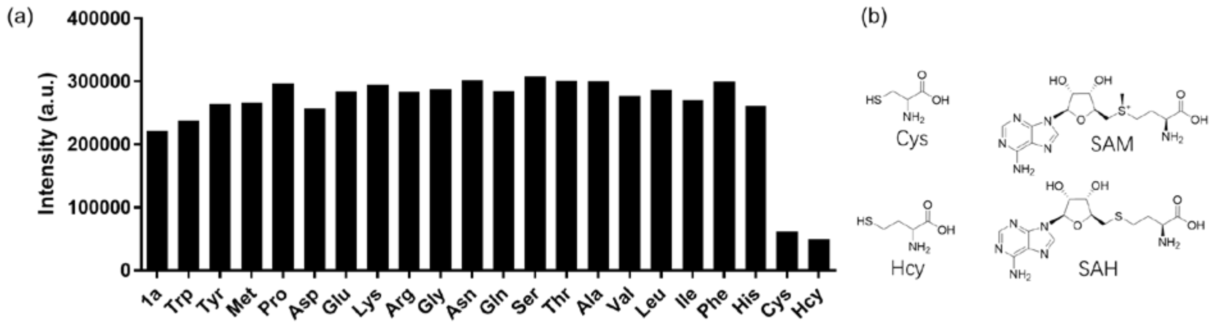


**Supplementary Fig. 5.** (a) Emission intensity of complex **Set7_1a** (20 µM) at 597 nm in Tris–HCl buffer (10 mM Tris–HCl, pH = 7.5, 10 mM KCl) with various amino acids (100 µM) at room temperature. (b) Chemical structures of Cys, Hcy, and co-factor SAM.

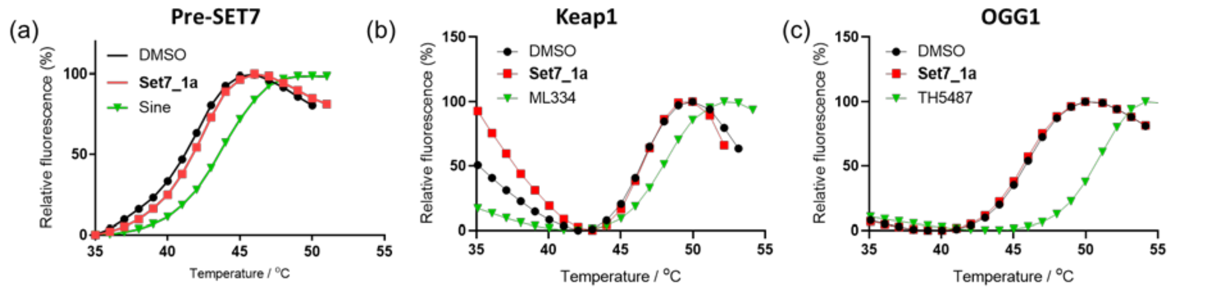


**Supplementary Fig. 6.** (a) FTS assay with PR-SET7 in the presence or absence of complex **Set7_1a** (10 μM) or positive control Sine (10 μM). (b) FTS assay with Keap1 in the presence or absence of complex **Set7_1a** (10 μM) or positive control ML334 (10 μM). (c) FTS assay with OGG1 in the presence or absence of complex **Set7_1a** (10 μM) or positive control TH5487 (10 μM).

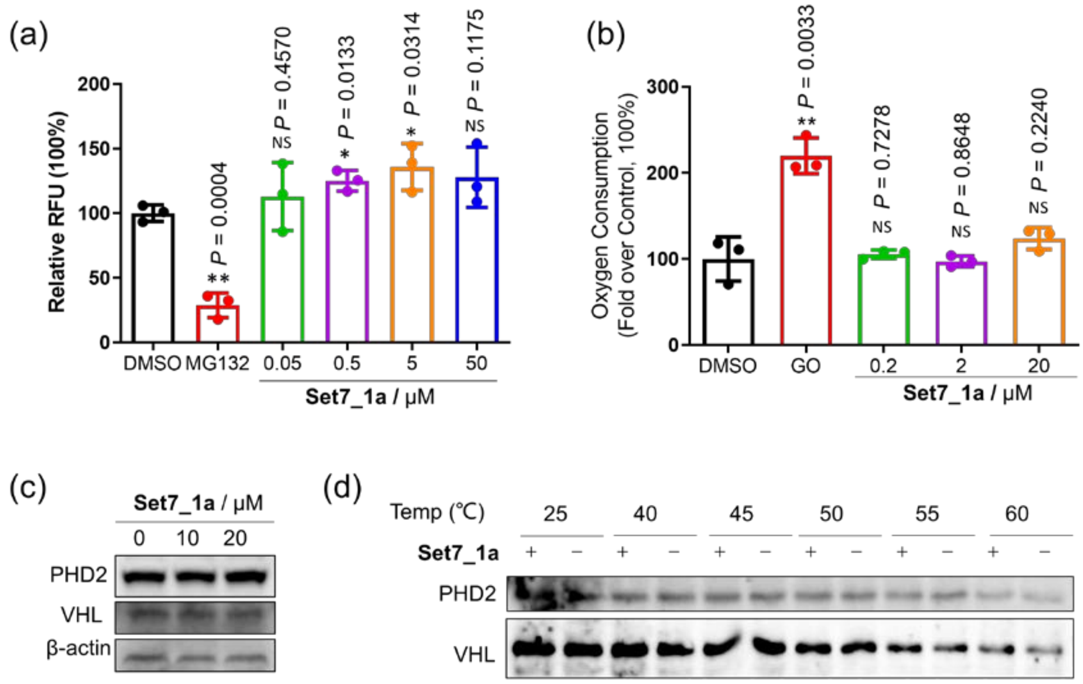


**Supplementary Fig. 7.** (a) The effect of complex **Set7_1a** against proteasome activity was monitored by using the commercial Proteasome Activity Fluorometric Assay Kit (BioVision Incorporated, CA, USA). Data are expressed as means ± SD ($n$ = 3). $P$ values were calculated using a two-sided t-test. *$P$ < 0.015, **$P$ < 0.01 vs. DMSO group. NS (not significant, $P$ > 0.05) vs. DMSO group. (b) The effect of complex **Set7_1a** towards oxygen consumption was monitored by using a commercial oxygen consumption rate assay kit (Cayman Chemical, MI, USA). Data are expressed as means ± SD ($n$ = 3). $P$ values were calculated using a two-sided t-test. **$P$ < 0.01 vs. DMSO group. NS (not significant, $P$ > 0.05) vs. DMSO group. (c) Immunoblotting assay to monitor the effect of complex **Set7_1a** on PHD2 and VHL levels. Hyperglycemia-induced HUVEC cells were treated with the indicated concentrations of complex **Set7_1a** for 12 h under hypoxic conditions. (d) Effect of complex **Set7_1a** on PHD2

and VHL thermal stabilities was monitored by using CESTA. Hyperglycemia-induced HUVEC cell lysates were treated with complex **Set7_1a** (10 μM) at room temperature for 30 min and then heated at different temperature ranging from 25 °C to 60 °C for 5 min. The protein samples were collected and detected by Western blotting using either PHD2 and VHL antibodies. NS: not significant.

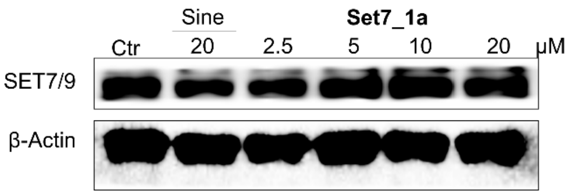


**Supplementary Fig. 8.** Effects of complex **Set7_1a** on the level of SET7/9 in HUVEC cells under hypoxic condition.

**MASS SPECTROMETRY REPORT**

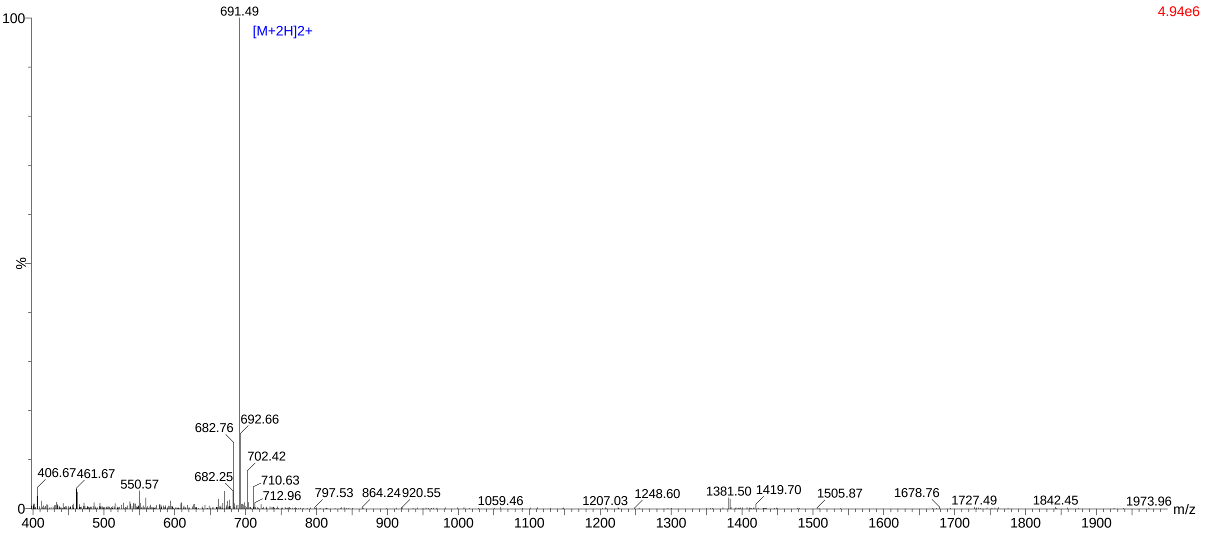


| | | | | | |
|---|---|---|---|---|---|
| Sample Description | | Instrument | Waters ZQ2000 | | |
| Analyzed date: | 2017-08-29 | Probe: | ESI | Probe Bias: | ＋4.5kv |
| Analyst: | YU | Nebulizer Gas Flow: | 1.5L/min | Detector: | 1.5kv |
| Sample: | RF-11 | CDL: | -20.0v | T. Flow: | 0.2ml/min |
| M.W.: | 1380.54 | CDL Temp.: | 250 °C | B. Conc.: | 50%H2O/50%ACN |
| Lot. No.: | P170821-VV538881 | Block Temp.: | 200 °C | | |

**Supplementary Fig. 9.** The synthesized peptide HIF-1α wild-type ($NH_2$-RSRRSKESEVF-COOH) peptides was confirmed by mass spectrometry.

**MASS SPECTROMETRY REPORT**

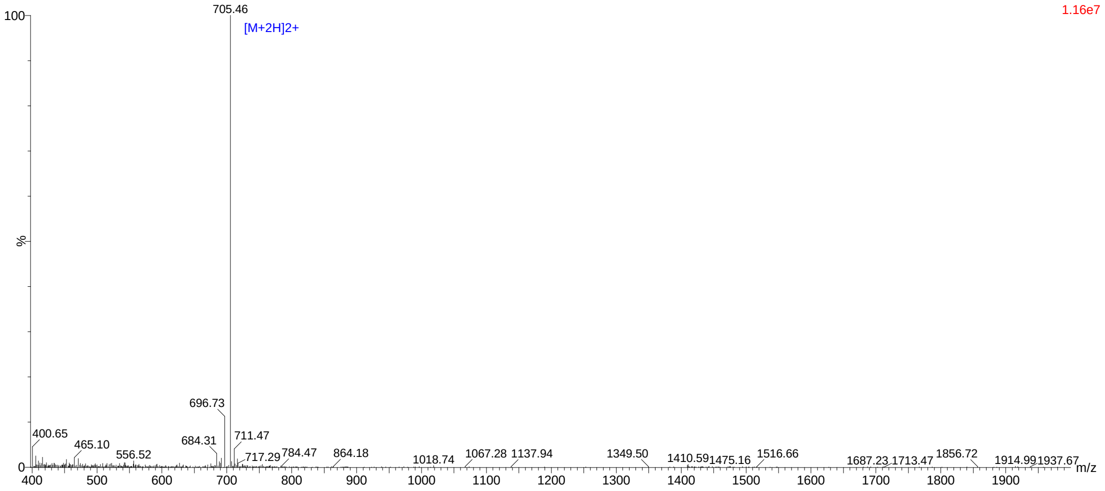


| Sample Description | | Instrument | Waters ZQ2000 | | |
|---|---|---|---|---|---|
| Analyzed date: | 2017-08-29 | Probe: | ESI | Probe Bias: | +4.5kv |
| Analyst: | YU | Nebulizer Gas Flow: | 1.5L/min | Detector: | 1.5kv |
| Sample: | RF-11 | CDL: | -20.0v | T. Flow: | 0.2ml/min |
| M.W.: | 1408.55 | CDL Temp.: | 250 °C | B. Conc.: | 50%H2O/50%ACN |
| Lot. No.: | P170821-VV538882 | Block Temp.: | 200 °C | | |

**Supplementary Fig. 10.** The synthesized peptide HIF-1α K32R ($NH_2$-RSRRSRESEVF-COOH) peptides was confirmed by mass spectrometry.

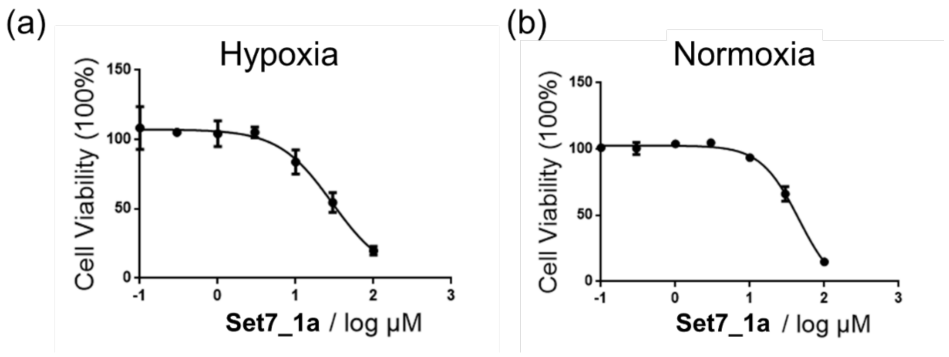


**Supplementary Fig. 11.** The cytotoxicity of complex **Set7_1a** *via* MTT assay. Hyperglycemia-induced HUVEC cells were treated with different concentration of complex **Set7_1a** for 48 h in hypoxic (a) and normoxic (b) conditions, respectively.

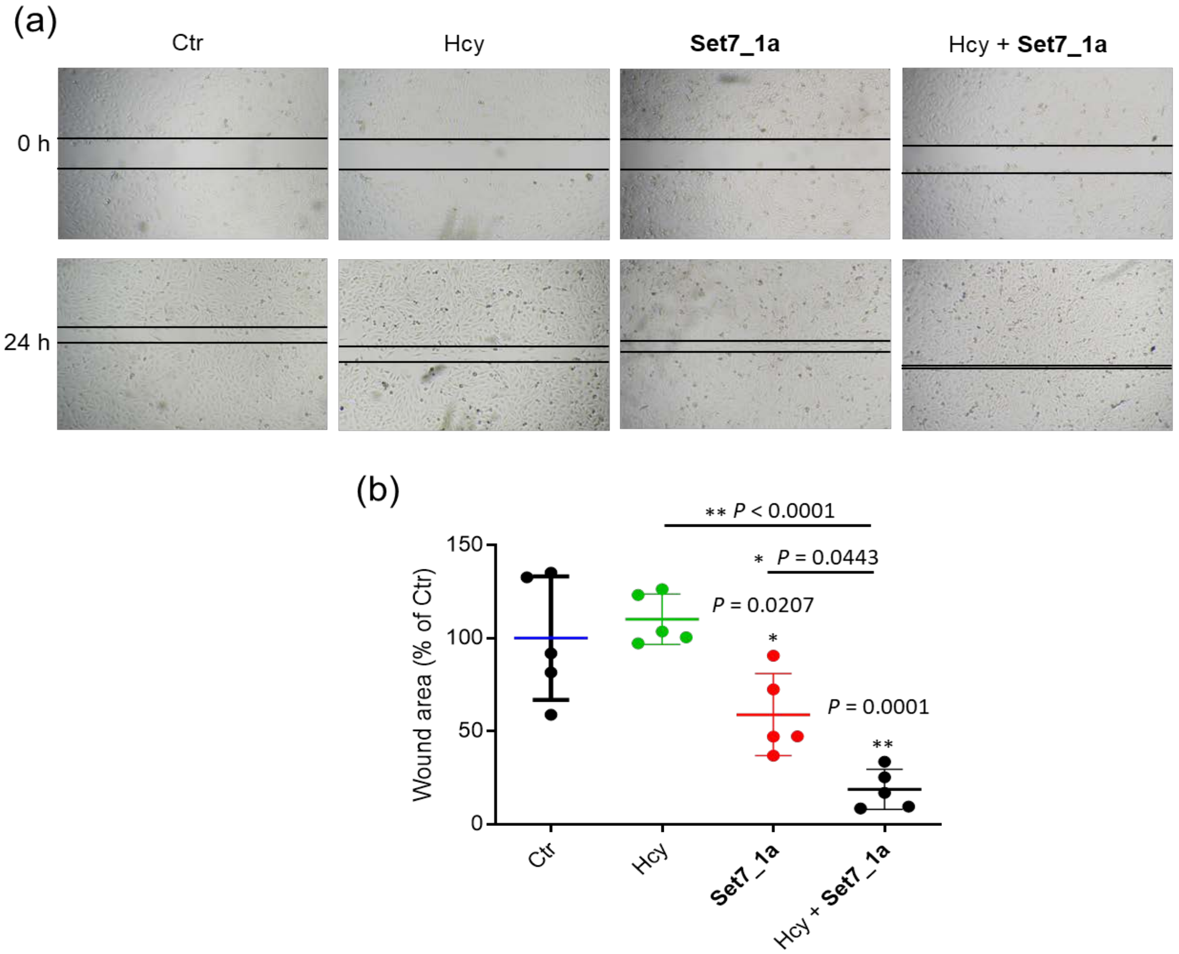


**Supplementary Fig. 12.** (a) Effects of Hcy on complex **Set7_1a**-induced HUVEC migration was assessed using an *in vitro* wound-healing assay. Monolayers of HUVECs were scratched and then incubated with complex **Set7_1a** (5 μM) with or without Hcy (100 μM) for 24 h in hyperglycemia-induced HUVEC cells in hypoxia. (b) Quantitation (right) analyses of the wound area.

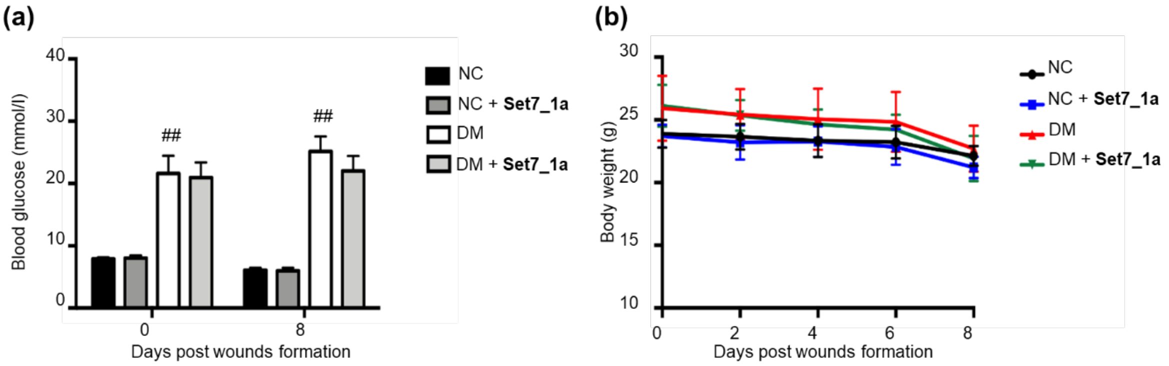


**Supplementary Fig. 13.** Effects of complex **Set7_1a** (50 mg/kg) on (**a**) blood glucose and (**b**) body weight in NC and DM mice. Data are expressed as means ± SD (n = 8). $^{\#\#}P < 0.01$ NC vs. DM.

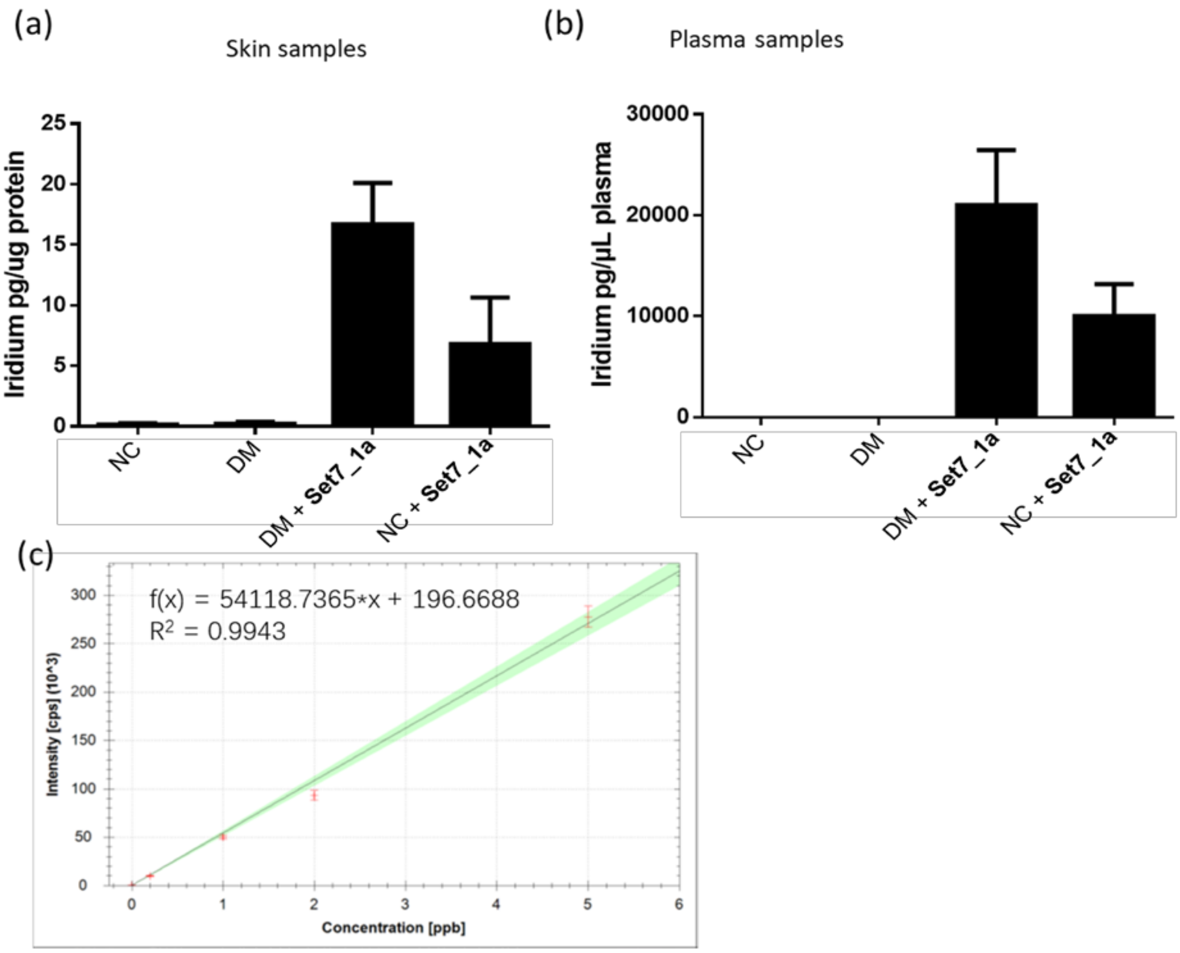


**Supplementary Fig. 14.** (a) ICP-MS assay for iridium content in skin samples. The vehicle and complex **Set7_1a** groups were intraperitoneally injected with vehicle (PEG 400: distilled water = 6:4, v/v) or 50 mg/kg complex **Set7_1a**, respectively every other day for 8 days, and skin and plasma samples were harvested. (b) ICP-MS assay for iridium content in plasma samples. (c)Standard curve of iridium.
